# Probe for simultaneous membrane and nucleus labeling in living cells and *in vivo* bioimaging using a two-photon absorption water-soluble Zn(ii) terpyridine complex with a reduced π-conjugation system†
†Electronic supplementary information (ESI) available. CCDC 939806, 939870 and 939871. For ESI and crystallographic data in CIF or other electronic format see DOI: 10.1039/c6sc02342h



**DOI:** 10.1039/c6sc02342h

**Published:** 2016-08-03

**Authors:** Xiaohe Tian, Qiong Zhang, Mingzhu Zhang, Kajsa Uvdal, Qin Wang, Junyang Chen, Wei Du, Bei Huang, Jieying Wu, Yupeng Tian

**Affiliations:** a Department of Chemistry , Key Laboratory of Functional Inorganic Material Chemistry of Anhui Province , Anhui University , Hefei 230039 , P. R. China . Email: xiaohe.t@ahu.edu.cn ; Email: yptian@ahu.edu.cn; b School of Life Science , Anhui University , Hefei 230039 , P. R. China; c Division of Molecular Surface Physics & Nanoscience , Department of Physics, Chemistry and Biology (IFM) , Linköping University , Linköping , 58183 , Sweden; d Biotechnology Centre , Anhui Agriculture University , Hefei , 230036 , China; e State Key Laboratory of Coordination Chemistry , Nanjing University , Nanjing 210093 , P. R. China

## Abstract

Small, biocompatible and water-soluble molecules with high two-photon absorption (2PA) cross-section values (*δ*) are in high demand for specific bioimaging applications. Here, two novel terpyridine derivative ligands with donor–acceptor (D–A) (**L1**) and donor–π–acceptor (D–π–A) (**L2**) models, and their corresponding Zn(ii) complexes are designed and characterized. It was found that the two-photon absorption cross section values (*δ*) in the near-infrared region (NIR, about 800 nm) are significantly enhanced for complexes **1** and **2** compared to their free D–A type ligand **L1**, while those of complexes **3** and **4** were greatly decreased relative to their free ligand **L2**, thus confirming that the smaller ligand (D–A type) displays a suitable Turn-ON fluorescence pair for two-photon fluorescence microscopy (2PFM). Firstly, the potential of simultaneously labeling a live cell plasma membrane and nucleus using complex **1** is demonstrated. In addition, live larval and adult zebrafish incubated with an optimal concentration of **1** demonstrated clear brain uptake. Lastly and importantly, using such a probe to visualize the blood–brain-barrier (BBB) capillary endothelial cells and penetrate the BBB into the central nervous system (CNS) intravenously in a mouse model is also explored.

## Abstract 2

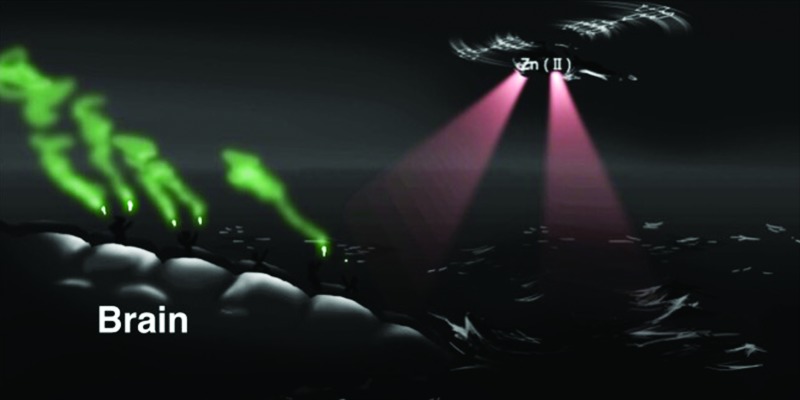
A two-photon absorption water-soluble Zn(ii) probe for simultaneous membrane nucleus live cell imaging and its potential for blood-brain barrier staining.

## Introduction

There has been considerable demand for developing materials with two-photon absorption (2PA) properties for biomedical applications.^1–4^ In particular, 2PA materials as luminescence probes allow imaging of thick tissue samples and specific intracellular organelles free from endogenous fluorophores by two-photon fluorescent microscopy (2PFM). However even in the best scenarios, the use of one probe to label two distinct organelles simultaneously by 2PFM has never been achieved before. It has been widely reported that extended and enlarged molecular D (electron-donor)–π–A (electron-withdrawing) [D–π–A] groups and their extended dipolar, quadrupolar and octupolar structures are endowed with high 2PA cross section values (*δ*).^4–6^ However, increased *δ* values often result in synthetic complications, large molecular weight and decreased water-solubility, which can lead to the employment of inappropriate solvents (*e.g.* DMSO) for living systems, high cytotoxicity, inaccurate subcellular targeting and obstacles to penetration through the biological barrier (*e.g.* aqueous barrier, plasma membrane and capillary endothelium).^2,4^ All of the above issues further limit their biological applications. Therefore, the balance between synthetic costs, *δ* values and biocompatibility is still a significant challenge needing to be urgently solved.

Recent studies have described the development of biocompatible, cell-permeable transition metal-based systems as attractive 2PA imaging agents. In particular, d^10^ metal Zn(ii) complexes generally possess lower toxicity and lower molecular weight than d^6^ (*e.g.* Ir^III^, Ru^II^) and d^8^ (*e.g.* Pt^II^) heavy metal complexes.^7–10^ Moreover, their high luminescence and electron dense cores render them suitable as dual function imaging probes for 2PFM and transmission electron microscopy (TEM).^11–13^ The investigations revealed that constructing novel metal complexes from suitable organic ligands and metal ions should give rise to fascinating photophysical properties. It is known that octahedral [M(tpy)_2_]core (tpy = 2,2′:6′2′-terpyridine) is a promising building block for the assembly of supramolecular architectures.^14–18^ While being kinetically inert (d^10^), the metal centers are less invasive and the complexes exhibit photophysical properties that can be tuned *via* substituents, being most readily introduced at the 4′-positions of the tpy ligands.^14–18^ Typically, these metal complexes possess multiple transitions such as metal-to-ligand charge transfer (MLCT) and ligand-to-ligand charge transfer in the visible region. The binding of protons or cations perturbs the charge density in the binding unit and subsequently induces optical signal switching between different energy states to achieve the desired optical responses. However, it is still a challenge to design and synthesize a Zn(ii) complex with low molecular weight, high water-solubility, biocompatibility and large 2PA cross sections in the near infrared range (700–900 nm).

**Fig. 1:**
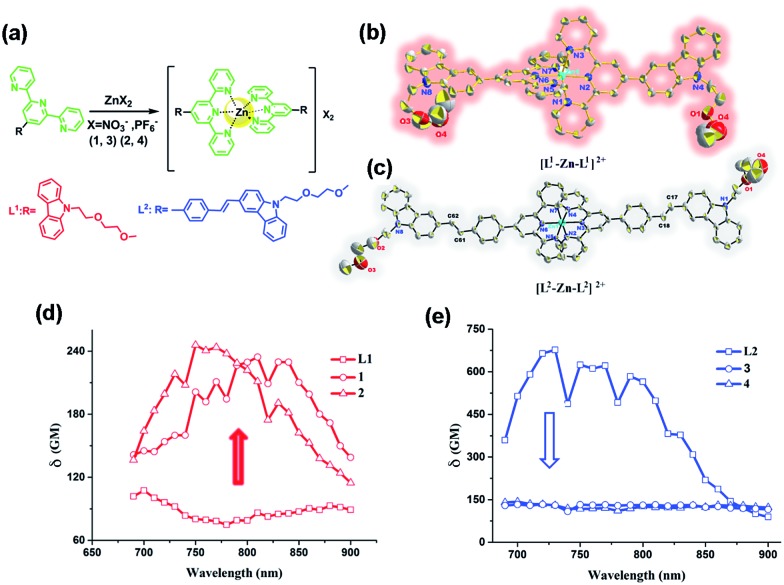
(a) Synthetic routes for all of the compounds. (b and c) The crystal structures of **2** and **4** (H atoms and PF_6_^–^ anions are omitted for clarity). (d) Two-photon cross-section of **L1**, **1** and **2** and (e) two-photon cross-section of **L2**, **3** and **4** in DMF with *c* = 1 mM at the optimal excitation wavelength.

Spurred on by this, we fabricated novel D–π–A and D–A type ligands in which the terpyridine ring acts as a tridentate chelate group and a carbazole moiety as a two-photon active moiety (Fig. 1a). This idea is based on the following considerations: (1) 2,2′:6′,2′′-terpyridine groups have an electron-withdrawing ability (acceptor) and their structural analogy with rich coordination chemistry has been extensively studied for their high binding affinity towards a variety of metal ions, resulting in interesting photophysical properties; (2) the carbazole group should be considered as a strong electron-donating group (donor); (3) a styryl unit as an extended π-conjugated system was inserted between the terpyridine and carbazole groups to construct **L2** for comparison with the σ-conjugated **L1**. (4) An extended polyether chain serves as a steric hindrance to diminish the intermolecular quenching effect, as well as having good solubility; it was also widely applied in *in vivo* drug delivery systems due to its immune-‘stealth-like’ behavior leading to increased blood circulation time and lower toxicity. Coordinating the designed ligand with Zn(ii) would be likely to lead to an enhanced 2PA response and fluorescence brightness due to the lack of d*–*d transitions. The selected complex in this study showed a unique live cell staining pattern and high biocompatibility in a vertebrate animal model.

## Experimental section

For detailed methods and approaches see the ESI.†

## Results and discussion

### Synthesis, characterization and crystal structure of the probes

**Scheme 1:**
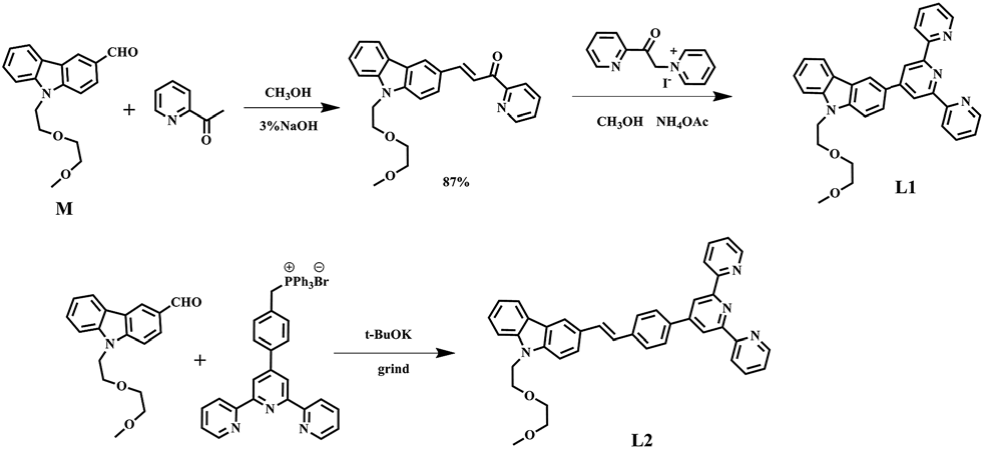
The synthesis routes of **L1** and **L2**.

The synthetic routes for the ligands (**L1**, **L2**) and their metal complexes (**1–4**) ([**L1**–Zn–**L1**](NO_3_)_2_ (**1**), [**L1**–Zn–**L1**](PF_6_)_2_ (**2**), [**L2**–Zn–**L2**](NO_3_)_2_ (**3**) and [**L2**–Zn–**L2**](PF_6_)_2_ (**4**)) are illustrated in Scheme 1 and Fig. 1a. As shown in Scheme 1, **L1** was prepared by a condensation reaction using 2-aceylpyridine and 3-acetyl-9-hexyl-carbaldehyde in the presence of ammonium acetate which afforded the title product in over 50% yield. **L2** was synthesized according to a series of improved methods.^19^ Orange crystals of **L2** were suitable for single crystal X-ray diffraction analysis and were obtained by slow evaporation of the remaining acetonitrile solution. Metal complexes were obtained through the reactions of corresponding ligands with zinc salts, which are obtained in acceptable yields of over 75%. The details of the characterization are presented in the ESI and Fig. S1.† The crystal structures of complexes **2** and **4** are shown in Fig. 1b and c. The crystal structure of ligand **L2** is shown in Fig. S2† and crystallography data of the compounds are given in Tables S1 and S2,† respectively.

As shown in Fig. 1b, the Zn(ii) center has been adopted into a distorted pseudo-octahedral geometry due to the coordination with a terpyridyl group. Because of the steric demand of the terpyridyl ligand, the N–Zn–N angles [N(2)–Zn(1)–N(3) = 75.47°; N(2)–Zn(1)–N(4) = 75.94°; N(5)–Zn(1)–N(6) = 76.00°; and N(6)–Zn(1)–N(7) = 75.63°] show deviation from the idealized values of 90° and 180°. The bond lengths of Zn–N [Zn(1)–N(2) = 2.068 Å; Zn(1)–N(3) = 2.192 Å; Zn(1)–N(4) = 2.189 Å; Zn(1)–N(5) = 2.167 Å; Zn(1)–N(6) = 2.070 Å; and Zn(1)–N(7) = 2.171 Å] are slightly shorter compared to those of the other terpyridyl Zn(ii) complexes.^20^ It is interesting that the carbazole plane also stacks with the [Zn(tpy)] plane with an interplanar angle of 22.82° and 16.52°, respectively, while the dihedral angle between the carbazole and terpyridine planes in the ligand is 20.15°. The planarity and the conjugated geometric configuration indicate that the ligand has a delocalized π-electron system, which is necessary for 2PA activity. The equatorial coordination sphere containing one Zn atom and six N atoms of complex **2** may facilitate the π-electron delocalization, thus causing an enhanced 2PA response for **2**. Relying on the structural study, as well as their photophysical data (Table S3, Fig. S3–S5†), it was found that the Lippert–Mataga plots of complexes **1–2** gave much larger slopes than that of the corresponding free ligand **L1**, which infers larger dipole moment changes for the complexes with photo excitation (as shown in Fig. S5†).^21^ The larger values of **1** and **2** indicate that the molecule in the excited state has an extremely polar structure, and significantly influences their nonlinear optical properties, including their 2PA activity.^22^

For complex **4** (Fig. 1c), the Zn(ii) center adopts a pseudo-octahedral geometry with pyridine rings occupying both the axial and equatorial positions. Complex **4** showed a little distortion with a deviation of 0.132 Å from the equatorial coordination plane formed by a zinc atom and six nitrogen donors. Two terpyridine units coordinated in the complex are almost vertical (87.23°). However, each carbazole and benzene plane in the complex is a wider margin of the twist angle (14.34°) away from the mean plane in the ligand (43.91°), presumably reducing steric interference. Comparing the free ligand **L2** with its corresponding complex, there is no significant difference in the bond lengths, angles and the distortion of the plane. The structural features suggest that the zinc ion does not influence the electron distribution in the ligand, and only extends the conjugated system. Based on the structural information and the photophysical properties (**L2**, **3** and **4**) (Tables S2 and S3, Fig. S3†), the polyether group is constrained to be coplanar with the benzene ring, which has been shown in crystal structures **L2** and **4** (Fig. S2† and 1c). The much larger Stokes shifts offer critical support for the PICT (planar ICT) mode that might lead to the decreased tendency in nonlinear optical properties, as well as their 2PA response.^23^

### Two-photon activity of the probes

2PA activities of all the compounds **L1**, **L2**, **1–4** are confirmed in the range of 700–900 nm in DMF and in water (DMSO : H_2_O = 1 : 9, Fig. S7†). The 2PA cross-section (*δ*_max_) of all the compounds (**L1**, **L2**, **1–4**) in DMF is presented in Fig. 1d and e. The *δ* intensities of 2PEF clearly exhibit the sequence of **1**, **2** > **L1**. The reasons are proposed as follows. First, the enhancement of *δ* was explained in terms of increased charge separation assisted by the terminal strong electron-donating groups. Second, coordinating to the Zn(ii) ion leads to an increased withdrawing ability of the terpyridine rings in the push–pull dipolar ligands and more π-electron contributions from the Zn^2+^ metal ions, which converts the ligand to a more strongly polarized D–π–A unit. This, therefore, more favors the ICT, causing an enhanced two-photon absorption^24^ which is also in line with DFT calculations. Third, more sublevels of Zn(ii) can be introduced into the energy hierarchy, which permits more allowed electronic transitions to take place. Lastly, we inferred that not only the electron donating ability of the terminal group impacts the 2PA behavior but also the static and steric bulk as well.^25^ In this work, the static and steric bulk of the electron-donating terminal group plays a major role in 2PA behavior. However, the *δ* values of **3** and **4** both decreased largely compared to those of their corresponding ligand **L2**. It is commonly known that the increase of the conjugation length leads to an increase of the 2PA activity.^4–6,26^ Consistent with this, in the present work, we found that an extended π-bridge reasonably increases the length of the conjugated chain and leads to prevention of electronic transition floating, which is in agreement with the observed decrease of the 2PA cross-section. This explanation is highly consistent with the results from both structural determinations and Lippert–Mataga plots (Table S3, Fig. S2, S5 and S6†).

### Cytotoxicity and cell localization behavior

Although **L2** possesses a large 2PA, its water solubility is poor due to its large extended conjugated length and molecular weight. However, comparing those complexes with their free ligands, **1** has a moderately active *δ* value and good water solubility, as well as higher quantum yield, longer lifetime, and smaller molecular volume, which stimulated us to further explore its potential application for biological imaging.

**Fig. 2:**
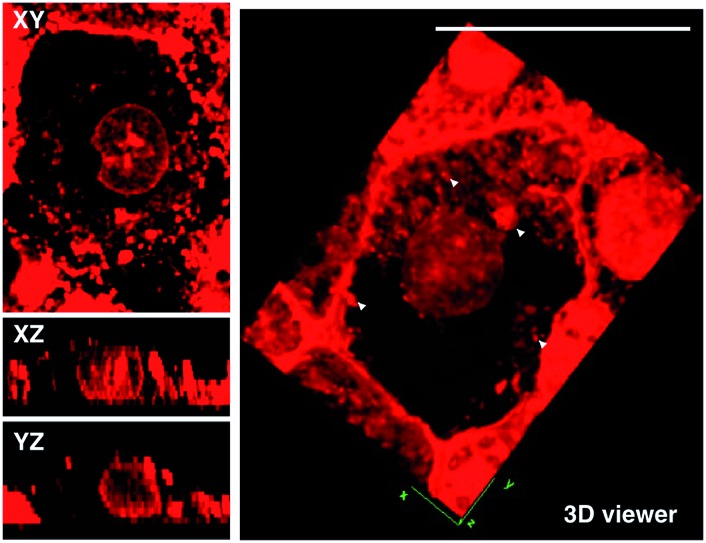
2D sections (Xy, Xz and Yz) of HepG2 cells stained by **1** and 3D rendering of a single HepG2 cell showing detailed uptake of **1** (10 μM, 30 min). Note: the white arrows might indicate intracellular vesicles. The scale bar represents 20 μm.

**Fig. 3:**
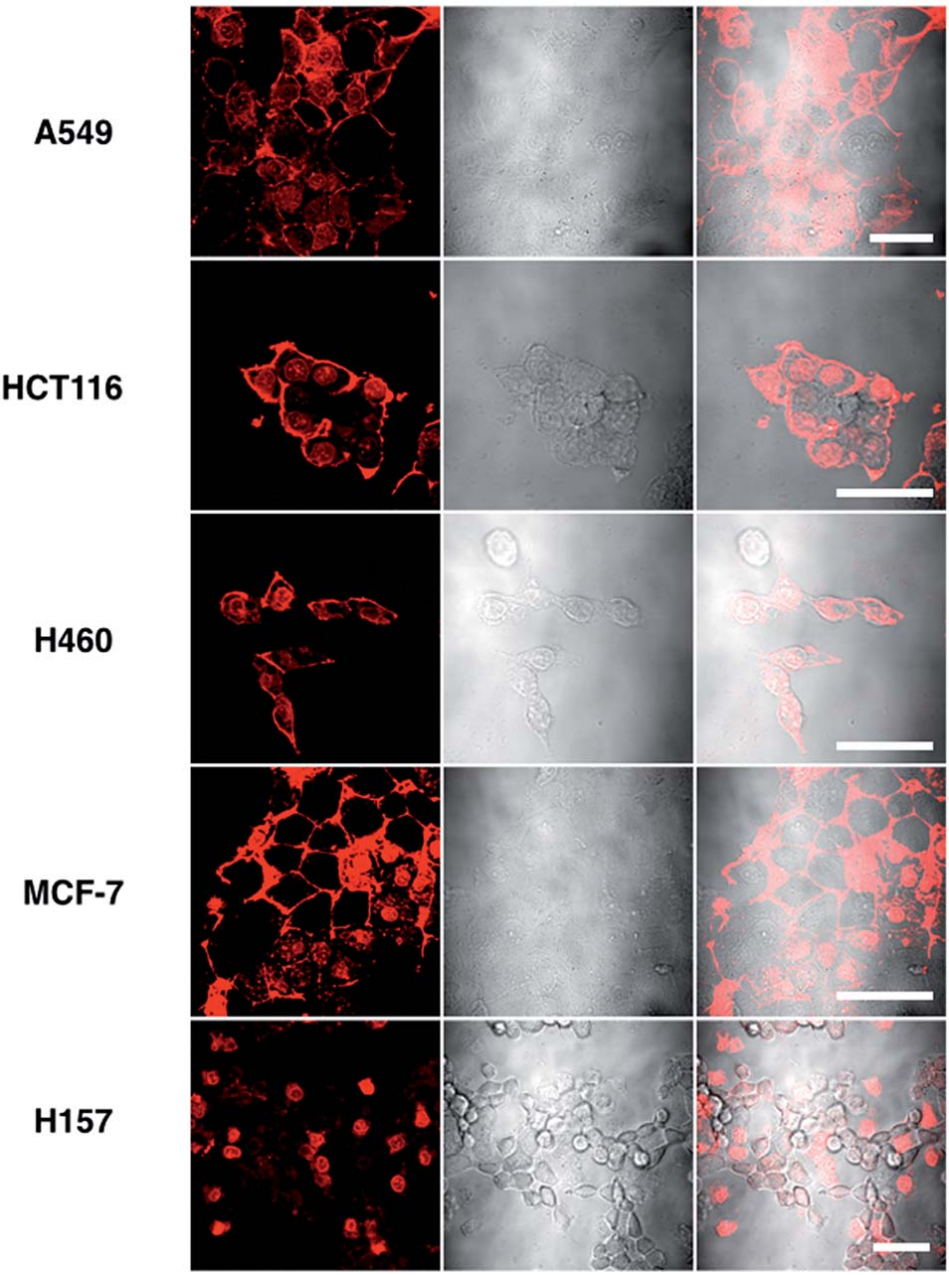
Images of 5 different types of living cancerous cells treated with **1** (10 μM, 30 min) merged with the DIC channel, the scale bars represent 20 μm.

**Fig. 4:**
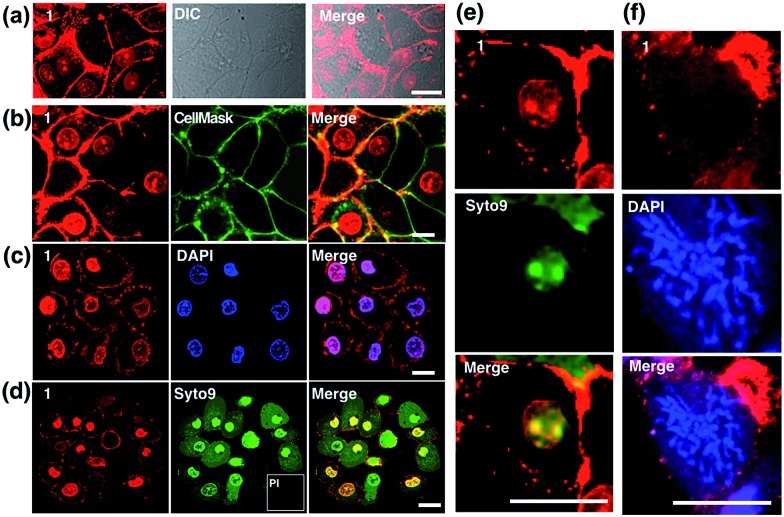
(a) Staining of HepG2 cells by **1** (10 μM, 30 min) showing the cellular uptake and *in cellulo* luminescence of **1** with a DIC micrograph. Confocal co-localisation studies of **1** with (b) CellMask, (c) DAPI and (d) Syto9 and Propidium Iodide (PI). (e) Higher resolution micrograph of an interphase cell uptake of **1** co-stained with Syto9. (f) Higher resolution micrograph of an interphase cell uptake of **1** co-stained with DAPI. The scale bar represents 20 μm.

MTT results of **1** demonstrated the extremely low toxicity of the complex (Fig. S8†). Therefore it is possible to investigate its biological properties as a 2PA image probe. To evaluate the cellular uptake of the complex, HepG2 (liver hepatocellular carcinoma) was used here as a cell model and incubated with **1** (10 μM in water, 30 min), and then imaged by 2PFM without fixation. The excitation wavelength applied was 820 nm for two-photon and 405 nm for one-photon, respectively. Fig. 4a showed that **1** displayed intense *in cellulo* luminescence with good photostability (Fig. S9†) in live cells after 150 s continued irradiation (820 nm, 30 minutes incubation). In particular the 2PA channel clearly showed a noise/autofluorescence-free micrograph compared to the 1PA channel (Fig. S10†). In contrast, the ligands (**L1** and **L2**) showed very weak cytosolic distribution (data not shown) and significant cytotoxicity (Fig. S8†); complex **2** displayed membrane staining with a weaker two-photon signal under the same microscopy parameters (Fig. S11†). Intriguingly, the dominant signal from complex **1** was located around the cell membrane and within the nucleus, whereas much weaker signals can be found within the cytosolic region. In particular, the bright dots within the nucleus are strongly believed to correspond to the nucleolus (refer to Fig. 4a). 2PFM Lambda scanning experiments indicated that the maximal emissions of **1** from different sources (nucleuses = 1, nuclear = 2, membrane = 3) were all located at approximately 530 nm, which suggested no red or blue shift for **1** after intracellular internalization (Fig. S12†). 3D-reconstruction micrographs confirmed plasma membrane, spherical nuclear and nucleolus labeling (Fig. 2). In addition, a few bright dots observed in the cytosol that closely associated with the membrane and nuclear region might imply the ongoing internalization of **1** from the cell boundary to the nuclear region through a micro-tubular-like structure. In parallel, the same treatment was applied on another five different types of cancer cells and a similar uptake pattern was observed on A549, HCT116, H460 and MCF-7 cells (Fig. 3). However, normal HELF cells (Human Embryo Liver Fibroblast) displayed diffusion of **1** throughout the cell cytosolic region and it was excluded from the cell nucleus (Fig. S13†). This might suggest that the membrane/nuclear uptake pattern of **1** was cancer specific.

### Colocalization studies of complex **1**

Colocalization experiments with the commercially available plasma membrane dye Cell Mask Deep-Red (Fig. 4b) and nuclear stain DAPI (Fig. 4c) have strongly suggested that **1** simultaneously targets the cell membrane and nucleus in living cells. Live/Dead assays with Syto9 (for RNA dye staining in live cells) and Propidium Iodide (PI, to stain nuclear DNA in dead cells) again confirmed high cell viability after the treatment (Fig. 4d). Furthermore, in magnified micrographs, a high degree of overlapping in the nuclear region between complex **1** and Syto9 can be obviously observed (Fig. 4e). It is known that eukaryotic nuclear regions mainly consist of abundant DNA, RNA and related structural proteins, wrapped by the nuclear membrane. It is noteworthy that in the dividing cells while the nuclear membrane disappeared (pro-metaphase, Fig. 4f), the nuclear signal of **1** completely disappeared once the chromosome and nucleolus disassembled, and this implied that the live cell binding sources of **1** in the nucleus are not DNA or RNA. To further investigate complex **1**’s preference *in vitro*, the interactions of **1** with numerous substances in the nucleus and plasma membrane, including various amino acids, proteins, liposomes, DNA, and RNA, were examined by fluorescent absorption (Fig. S14†). Notably, obvious luminescence enhancement was observed between **1** and l-cysteine and luminescence reduction was shown with basic amino acids including histidine, arginine and lysine, suggesting interactions between **1** and these amino acids *in vitro*. These results imply that the living cell membrane and nuclear staining might be due to the fluorescent quenching and enhancement while the metal complex binds with the residues within alkaline amino acid rich protein and l-cysteine rich protein, respectively, *via* a specific cell entry pathway.^27^

### Use of **1** as a transmission electron microscopy agent

**Fig. 5:**
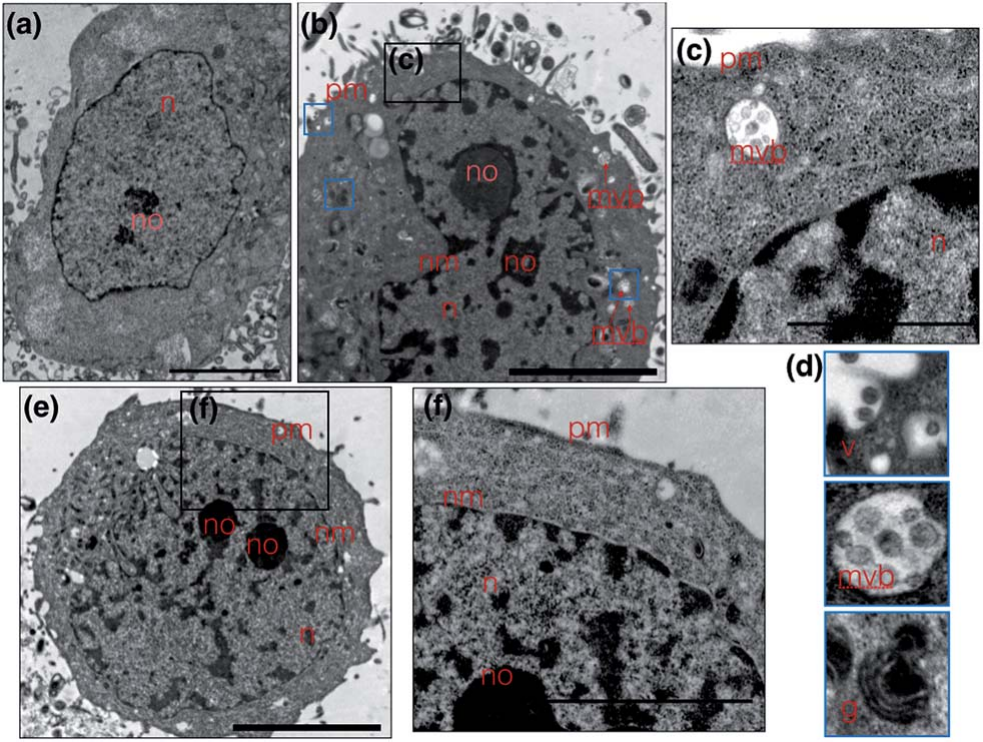
(a) Untreated HepG2 cells stained solely with osmium tetroxide; (b) TEM micrograph of HepG2 cells incubated with **1** and stained with osmium tetroxide clearly showing better nuclear contrast. (c and d) Zoomed in TEM micrograph from selected regions from (e) clearly showing multi-vesicular bodies and on-going endocytotic membrane system. (e) HepG2 cell uptake of **1**, where compound **1** is used directly as a contrast agent and (f) the higher resolution micrographs showing strong plasma membrane and nuclear contrast. The scale bar represents 5 μm. Abbreviations: n = nuclear, no = nucleolus, nm = nuclear membrane, pm = plasma membrane, mvb = multi-vesicular body, v = vesicles, g = Golgi apparatus.

Metal complexes are able to scatter the electron beam in TEM due to their high electron density and intracellular local accumulation.^11–13^ Therefore, the contrast is enhanced in this way similarly to gold/silver particles and osmium tetroxide. We then explored **1** as a dual function image probe using TEM. Compared to control cells stained solely with osmium tetroxide (Fig. 5a), a phospholipid contrast reagent that has been widely used in electron microscopy, clear subcellular membrane structures including mitochondria, intracellular vesicles, plasma membrane and nuclear membrane in treated cells were observed. When HepG2 cells were incubated with **1** and co-stained with osmium tetroxide, it was found that the cell nucleus especially the nucleolus displayed much better contrast due to the accumulation of **1** (Fig. 5b). It is interesting to note that a number of multivesicular bodies (MVB, Fig. 5b arrows and Fig. 5c) can be clearly observed and **1** was apparently encapsulated. Higher resolution TEM micrographs (Fig. 5d) selected from Fig. 3b again present the endocytotic membrane compartment^28^ including membrane-engulfing vesicles, multi-vesicular bodies and Golgi apparatus all with relatively greater contrast. General endocytosis traditionally can be classified into pinocytosis and phagocytosis, while the former exists in almost all eukaryotic cells ubiquitously. Since the higher resolution EM micrographs (Fig. 5d) displayed specific folding/binding events at the plasma membrane, as well as sorting trafficking multi-vesicular bodies, these all strongly implied that an endocytotic pathway is involved in the cellular uptake and sorting of **1**. Notably, as highlighted in Fig. 5e, when HepG2 cells were solely incubated with **1** without co-staining with osmium tetroxide or any other agents, less contrast was observed in the intracellular membrane compartments as expected. In contrast, a much higher concentration of **1** was found on the plasma membrane and in the nucleus/nucleolus (Fig. 5f), which is in agreement with the phenomenon exploited using 2PFM.

### Possible cell entry mechanism of **1**

**Fig. 6:**
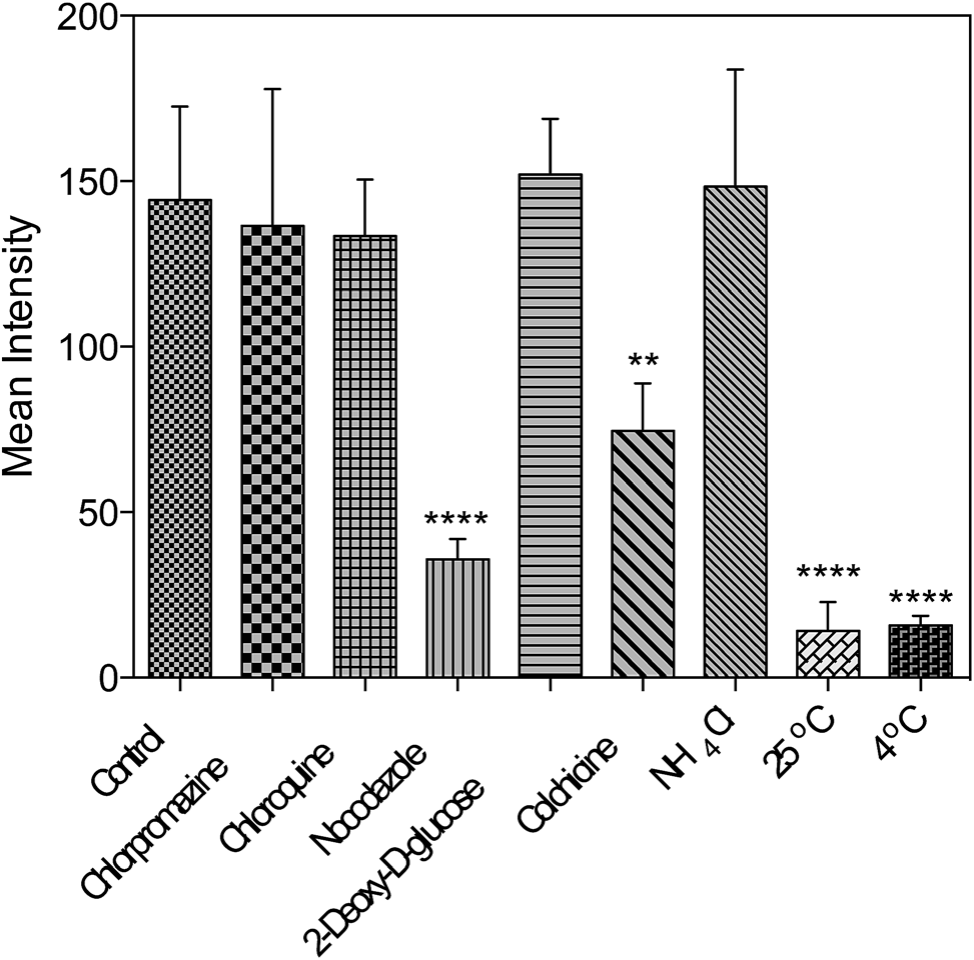
Fluorescent intensity analysis for living HepG2 cells treated with **1** and different inhibitors. One-way ANOVA was used for statistical analysis, *p* < 0.005, error bars: SEM.

Further studies were performed either under low incubation temperature or with several well-documented inhibitors^29,30^ (Fig. 6 and S15†) to understand the cell entry mechanism of **1**. It is suggested that the possible uptake mechanism of **1** is the hijacking of endosomal trafficking on the intracellular microtubules.^31^ This result is in good agreement with the 2PFM and TEM results, as well as the micrographs taken with the pre-fixed and pre-permeabilized HepG2 cells after incubation with **1** at 37 °C. It was demonstrated that once the subcellular membranes/membrane proteins were defunctionalized due to the fixation, **1** no longer displayed the specificity towards the plasma membrane and nucleus. Instead, a generalized staining pattern that was weakly diffuse across the whole cell was clearly observed (Fig. S16†). This might also explain the quenching effect of **1** in the cytosolic region, since the acidic endosome/lysosome was involved during the endocytotic pathway and subsequently triggered the intensity reduction of **1** (Fig. S14†).

Concerning all of the results above, the potential mechanism can be proposed as: **1** binds with the plasma membrane and interacts with specific membrane transport proteins, enters cells *via* the energy-consuming processes, namely endocytosis, and accumulates to a great extent in the cell nucleic region. However, the actual binding protein(s) and transport mechanism that induce the membrane/nuclear simultaneous labeling are still under investigation. Nonetheless, to the best of our knowledge, probes that can simultaneously stain the plasma membrane and cell nucleus under 2PFM and TEM do not exist so far.

### Internalization of **1** with a larval zebrafish model

**Fig. 7:**
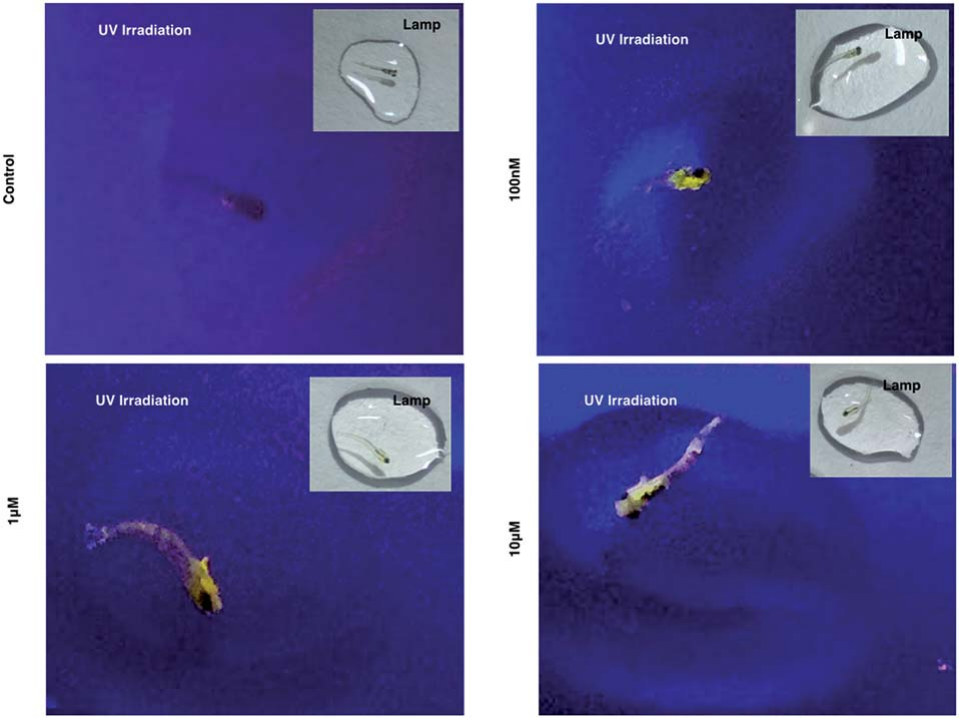
Larval zebrafish treated with **1** and imaged under UV light and lamplight.

**Fig. 8:**
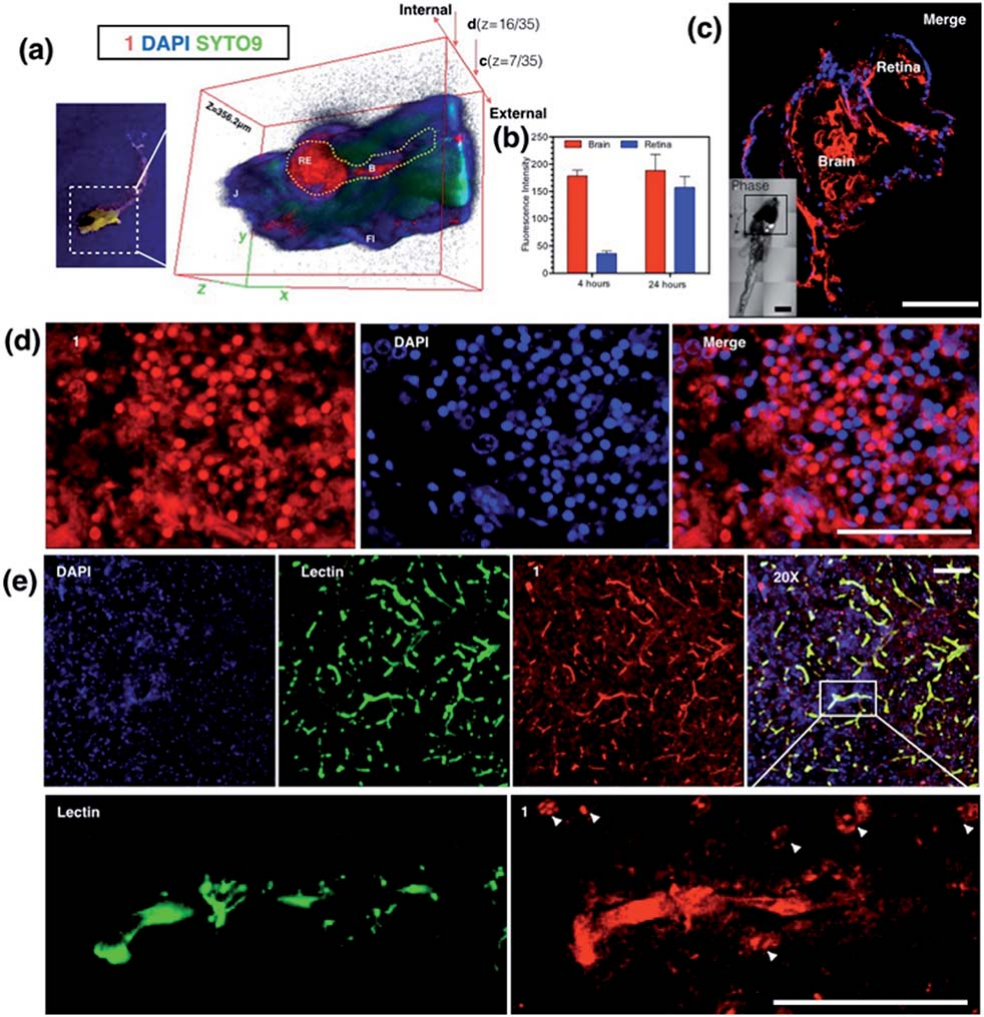
(a) Anaesthetised larval zebrafish incubated with **1** (100 nM) for 24 hours under UV light (left) and 3D micrographs (thickness = 356.2 μm) from two-photon confocal microscopy co-stained with DAPI, Syto9 and propidium iodide. (b) Fluorescence intensity analysis of the uptake of **1** in larval zebrafish brain and retina at 4 and 24 hours. (c) Whole brain region imaging of stained larval zebrafish by **1** co-stained with DAPI, insert: DIC tile scanning of the imaged larval zebrafish. (d) Brain sections of stained adult zebrafish by **1** co-stained with DAPI. (e) *Ex vivo* assessment of **1** following i.v. injection in mice co-labeled with DAPI and Alexa488-lectin, and zoom in (200×) micrographs showing the detail of the brain capillaries imaged by confocal laser scanning microscopy, with strong internalization of **1** by the brain endothelium and CNS cells at the nuclear and plasma membrane. The scale bar represents 200 μm. Error bars: SEM, *n* = 3. Abbreviations: Re = retina, B = brain, J = jaw, FI = fin.

As **1** has been successfully utilized as a convenient and non-invasive 2PA probe in living cells with superb photostability and cell permeability compared to its insoluble ligand, it also showed good deep tissue penetration on a solid tumor model using HepG2 multi-cell spheroids and displayed membrane and nuclear labeling (Fig. S23†), we were then motivated to evaluate its potential in small animal labeling. Zebrafish^32^ were chosen and a larva survival experiment was performed firstly to ensure the low cytotoxicity of **1** for living vertebrate animals. Zebrafish larvae (post 7 days, number = 50/group) were incubated with **1** (100 nM, 1 μM, 10 μM) for a 10 day period of time. The high survival rate indicated the extremely low toxicity of **1** for living zebrafish larvae (Fig. S17†). To primarily test the diffusion of **1** into living organisms, representative zebrafish larvae were chosen from each group at a time point of day 1 (∼24 hours incubation), anaesthetized (level-III) using MS222 (ref. 33) and imaged under lamplight or UV-light. In the control group, there was no irradiation with the fish upon exposure to UV light, whereas in the group treated with **1**, strong florescence was emitted throughout the fish body and particularly dominated the head compartment (Fig. 7). These experiments have further revealed that **1**, as a 2PA probe, is non-toxic towards zebrafish larvae. Subsequently **1**-labeled anaesthetized larval zebrafish were moved to 2PFM to examine the precise location in the fish’s body under a two-photon laser (*E*_x_ = 850 nm, *E*_m_ = 450–550 nm). The 3D micrograph in Fig. 8a (and Fig. S18†) shows that **1** can be found throughout the fish’s body up to ∼350 μm in depth. It is also interesting to note that apparently a large amount of **1** was located in brain and retina system. Fluorescent intensity analysis from two time points, 4 hours and 24 hours, indicates that **1**'s signal in the brain remains unchanged while the signal in the retina increased over time (Fig. 8b), which highly suggests that the possible spread direction of **1** is from the brain to the retina. It is in line with expectations as in the vertebrate central nervous system, the brain is physically associated with the retina system *via* fabric optic nerves.^34^ Further magnified micrographs from zebrafish larvae incubated for 6 hours with **1** clearly displayed significantly higher intensity in the brain region (mid-brain) and clear internalization by parenchymal cells (Fig. 8c).

### Internalization of **1** with an adult zebrafish model

The diffusion capability of **1** into thicker tissue was also evaluated using adult zebrafish (24 hours, 100 nM). The brain section again presented intense 2PA fluorescence (Fig. 8d). The colocalization experiment with DAPI has further revealed that abundant **1** clearly targets the brain cell nucleus, while an intense 2PA fluorescence signal was also found in the brain extracellular space. These results suggested the efficient diffusion ability of **1** in the brain system and efficient internalization in central nervous system (CNS) cells in such small living vertebrate animals. Similar spreading and intracellular uptake patterns were also observed in retina sections (Fig. S19†). However, the distribution of **1** is restricted in the retina system and the staining is limited to the inner nuclear layer.^35^ Clearly with its non-invasiveness, **1** has been employed here as a probe in small animals which has successfully labeled both larval and adult living zebrafish under two-photon excitation. In particular, it has been found that **1** has higher affinity for staining the brain system both extracellularly and intracellularly.

### *In vivo* assessment of **1** into the CNS in a mouse model

Later, we were further motivated to explore its biodistribution in a more complicated mammalian model, as **1** displayed intense 2PA luminescence and great organism penetration in zebrafish. Here mice (*n* = 3) received 2 i.v. injections in 24 hours (see experimental methods for details) *via* the tail vein. None of the treated mice displayed obvious side effects or died after injection. The internalization and subsequent biodistribution of **1** were then examined in the brain, liver and kidney, while the cell nucleus and capillary endothelium were stained with DAPI and Alexa488 conjugated-lectin, respectively. It is not surprising that a relatively higher level of **1** was detected in the liver (Fig. S20†) and kidney (Fig. S21†), as it is known that one of the main functions for such organs is to metabolize drugs or toxins externally and internally. Consistent with the above cell and zebrafish studies, in capillaries including the blood–brain barrier which is made of brain capillary endothelial cells, a very high degree of internalization of **1** has been observed (Fig. 8e) 24 hours after injection. Zoom-in micrographs (Fig. 8e and S22†) demonstrated that cells closely associated with brain capillaries (indicated by white arrows) also display the accumulation of **1**, suggesting the positive uptake of **1** in pericyte and astrocyte end-feet within the neuron vascular unit (NVU).^36^ Additionally, **1** was not only highly localized to the brain capillary endothelium, but also internalized by the CNS cell nucleus (as indicated by white arrows) in deeper tissues in the brain parenchyma, consistent with previous *in cellulo* and zebrafish tests, which again has proved its high affinity with nucleic proteins.

A few examples of nuclear targeting zinc terpyridine complexes have been found in the literature; however, those studies were restricted to cell level.^37,38^ It is known that blood–brain barrier (BBB) endothelial cells express extremely tight ‘tight junctions’ that lead to severe restriction of paracellular transport, and it was considered the most important target for designing new routes to facilitate drug or imaging agent delivery into the CNS,^39^ whereas most of the existing candidates fail to do so. Here this study showed that nontoxic **1** with 2PA fluorescence showed good penetration into the CNS through the BBB and stained brain parenchymal cells. We therefore anticipate that further modification of **1** to increase the BBB specificity (*e.g.* peptide-conjugation Rabbit virus glycoprotein^40^) might lead to a potential dual/multifunctional agent for CNS therapeutic and imaging applications.

## Conclusions

In conclusion, four novel Zn(ii) complexes based on two terpyridine chromophores containing carbazole groups as ligands (**L1**, **L2**) have been synthesized and confirmed by single crystal X-ray diffraction analysis. A significant advance of our current study is that we have successfully revealed that two different conjugation bridges (π and σ) of the terpyridine ligands can arouse largely different optical properties, which were confirmed both experimentally and theoretically. Considering the easier electron contribution from the metal ions, it is inferred that an extended conjugation bridge, for instance in the σ-bridge system, may favor the ICT and thus cause significantly enhanced two-photon absorption. Intriguingly, **1** can be used as an imaging probe to simultaneously light up the plasma membrane and nucleus in living cells *via* an endocytotic pathway. Effective diffusion in the brain compartment in larval and adult zebrafish was observed to a depth of over ∼300 μm. In particular, in a mouse model, intravenously administered **1** displayed high affinity with the BBB and showed good penetration into the brain parenchyma. In fact, from the images obtained in this study, especially those in mammalian tissues, we are enthusiastic that, in the future, class-specific small molecular probes based on terpyridine groups with further modification/functionalization could be discovered.
