# Supplementary material for: Probe for simultaneous membrane and nucleus labeling in living cells and in vivo bioimaging using a two-photon absorption water-soluble Zn(ii) terpyridine complex with a reduced π-conjugation system
Source: Chem Sci. 2016 Aug 3;8(1):142–9. doi: 10.1039/c6sc02342h (PMC5308395; doi:10.1039/c6sc02342h)
Supplement: Supplementary file 1 [file SC-008-C6SC02342H-s001.pdf]

Electronic Supplementary Material (ESI) for Chemical Science.
This journal is © The Royal Society of Chemistry 2016

Supporting Information

Spontaneously Probe Membrane and Nucleus *in Vitro* and CNS *in Vivo* Using A Water Soluble Zn(II) Terpyridine Complex with Two-photon absorption

Xiaohe Tian [ab‡], Qiong Zhang[ac‡], Mingzhu Zhang[b‡], Kajsa Uvdal[c], Qin Wang[d], Junyang Chen[b], Wei Du[a], Bei Huang[b], Jieying Wu[a], and Yupeng Tian[ad*]*

26th September 2016

Note added after first publication: This Supplementary Information file and the associated CIF file replace those originally published on 17th August 2016, which contained incorrect crystal structure data for CCDC 939806.

Experimental sections

Materials and Apparatus

All chemicals and solvents were dried and purified by usual methods. Elemental analysis was performed with a Perkin–Elmer 240 analyzer. IR spectra (4000–400 cm–1), as KBr pellets, were recorded on a Nicolet FT–IR 170 SX spectrophotometer. Mass spectra were obtained on a Micromass GCT-MS Spectrometer. ^{1}H and ^{13}C NMR spectra were recorded on a Bruker AV 400 spectrometer with tms as internal standard.

Synthesis and characterization

The synthetic routes for the ligands (**L1** to **L2**) and their metal complexes were illustrated in **Figure S1**. Through the condensation reaction and Solvent-free Wittig reaction, **L1 to L2** was obtained in high yields. Both the ligands were purified over recrystallization. The structures of ligands and their metal complexes were characterized by IR, ^{1}H NMR, ^{13}C NMR spectra and elemental analyses. ^{1}H-NMR spectra of the complexes display well resolved peaks, ranging from 1.29 to 9.43 ppm. Compared with those in free ligands, their positions in the complexes were shifted to lower fields. Additionally, four crystal structures were further confirmed by single crystal X-ray diffraction analysis.

9-(2-(2-ethoxypropoxy)ethyl)-9H-carbazole-3-carbaldehyde (**M**)

As seen in Figure S1, to a solution of carbazole (3.34 g, 20 mmol) in DMF (80 mL) at 0 °C was added NaH (0.72 g, 30 mmol). After heating at 80 °C for 1.5 h, 1-chloro-2-(2-ethoxypropoxy)ethane (4.37g, 24 mmol) was added dropwise. The resulting mixture was kept at 80 °C overnight. After cooling down to 0 °C, the reaction mixture was carefully quenched with water and extracted with ethyl acetate three times. The combined organic phase was washed with water and brine. Then the organic layer was dried over anhydrous sodium sulfate and the solvent was removed. The residue was purified by silica gel chromatography using petroleum ether and ethyl acetate as eluent (EA : PE = 1 : 3) to afford alkylated carbazole 1, and then, 6.3 g (0.02 mol) of 1 was reacted with 7.3 g (0.1 mol) of dimethylformamide in the presence of 27 g (0.2 mol) of phosphorus oxychoride by mixing the first two reactants and adding the latter dropwise with stirring while cooling the reaction vessel in an ice bath. The mixture was

heated to reflux for about 1 h, and then poured into ice-water, neutralized with 20% sodium hydroxide, and then the product was obtained by filtration. The crude product was purified through column chromatography on silica gel using dichloromethane and petroleum ether mixed solution (1:3) as eluent. Pale yellow liquid with yield 85% were obtained.^{1}H NMR (CDCl3, 400 MHz) δ (ppm) = 3.30 (s, 3H), 3.42(q, 2H), 3.53(q, 2H), 3.92(t, J = 6.0 Hz, 2H), 4.56 (t, J = 8.0 Hz, 2H), 7.35(t, J = 5.6 Hz, 1H), 7.51-7.59 (m, 3H), 8.01(d, J = 7.6 Hz, 1H), 8.16(d, J = 7.6 Hz, 1H), 8.61(s, 1H), 10.11(s, 1H). ^{13}C NMR ($CDCl_3$, 150 MHz): 191.03, 139.81, 135.74, 126.54, 126.23, 125.11, 121.82, 121.70, 121.46, 119.83, 113.52, 109.62, 71.62, 71.24, 70.45, 70.10, 59.36, 54.91. IR (KBr, cm^{-1}): 3438 (w), 3056(vw), 2871(m), 1688(vs), 1590(s), 1474 (s), 1130 (s), 1114(m), 801(m),741(m). MALDI –TOF –MS: *m/z*, 341.2 [M^+, 100%].

Preparation of N-(methoxyethoxy)ethyl-3-{2-[4-(2, 2' : 6', 2'' - terpyridine]carbazole (L1) *t*-BuOK (1.6 g, 13.9 mmol) was dissolved in 30 mL THF, and 2-aceylpyridine (1.1 g, 8.95 mmol) was added to the solution. After the reaction mixture was stirred at room temperature for 2 h, 9-(2-(2-methoxyethoxy)ethyl) -9H- carbazole -3-carbaldehyde[1] (1.18g, 4 mmol) was added dropwisely. After the reaction mixture was stirred at room temperature for 8 h, the ropy mixture was added to a stirred solution of ammonium acetate (12.0 g, excess) in ethanol (250 mL). The reaction was heated at reflux for 5 h, affording a dark red solution. Cooled to the room temperature, bright yellow product was crystallized from the filtered solution. The yellow granular product was recrystallized from ethanol and dried in vacuum to give pale yellow crystals **L1** with yield 53%.^{1}H NMR ($CDCl_3$, 400 MHz) δ (ppm) = 3.34 (s, 3H), 3.46 (q, 3H), 3.55 (q , 2H), 3.93 (t, J = 5.9 Hz, 2H), 4.57 (t, J = 5.9 Hz, 2H), 7.31 (dd, J = 8.0, 3.9 Hz, 1H), 7.42 - 7.35 (m, 2H), 7.51 (d, J = 3.6 Hz, 2H), 7.60 (d, J = 8.5 Hz, 1H), 7.91 (t, J = 7.7 Hz, 2H), 8.07 (d, J = 8.5 Hz, 1H), 8.24 (d, J = 7.7 Hz, 1H), 8.70 – 8.66 (s, 1H), 8.75 - 8.70 (m, 2H), 8.79 (d, J = 4.6 Hz, 2H), 8.89 (s, 2H). ^{13}C NMR ($CDCl_3$, 150 MHz): δ (ppm) = 155.47, 155.23, 150.38, 149.24, 140.99, 140.80, 137.33, 128.26, 126.09, 124.54, 124.35, 122.95, 122.27, 120.92, 120.78, 119.27, 118.79, 117.88, 110.39, 109.81; 71.23, 70.50, 69.78, 68.80, 58.02; IR (KBr, cm-1): 3445 (m), 3063(vw), 2918(w), 1581(s), 1467 (s), 1123 (m), 795(m), 750 (m). MALDI–TOF–MS: *m/z*, 500.2 [M^+, 100%].

Preparation of N-(methoxyethoxy)ethyl-3-{2-[4-(2,2':6',2''-terpyridin-4'-yl) phenyl] ethenyl}-carbazole (L2) 9-(2-(2-methoxyethoxy)ethyl)-9H-carbazole-3-carbaldehyde(0.89g,3 mmol), 4-(2,2':6',2''-terpyridyl-4')-benzyl triphenyl phosphonium bromide[2] (1.75g, 3 mmol) and *t*-BuOK (1.3 g, 12.3 mmol) were placed into a dry mortar and milled vigorously for about 20 mins, and monitored by TLC until reaction completion. The mixture was dispersed in 100 mL ethanol. The residual solid was filtered and recrystallized from water/ethanol, giving pale yellow crystals of L2 with yield 72%. ^{1}H NMR (CDCl3, 400 MHz) δ (ppm) = 3.34(s, 3H), 3.46(q, 2H), 3.53(q, 2H), 3.91(t, J = 6.0 Hz, 2H), 4.54(t, J = 6.0 Hz, 2H), 7.23(d, J = 16.0Hz, 1H), 7.30(s, 2H), 7.41(m, 3H), 7.46(s, 1H), 7.50(d, J = 3.6Hz, 2H), 7.72(t, J = 6.6 Hz, 3H), 7.93(t, J = 6.8Hz, 2H), 7.98(d, J = 8.0Hz, 2H), 8.15(d, J = 7.6Hz, 1H), 8.27(s, 1H), 8.73(d, J = 8.0Hz, 2H), 8.79(d, J = 4.4Hz, 2H), 8.84(s, 2H). ^{13}C NMR ($CDCl_3$, 150 MHz): δ (ppm) = 155.6, 154.98, 149.31, 148.92, 140.69, 140.24, 138.99, 137.43, 135.53, 130.69, 128.04, 127.18, 126.96. 125.81, 124.68, 124.49, 122.49, 122.15, 120.91, 119.09, 118.69, 117.47,

109.91, 109.81, 71.24, 69.76, 68.82, 58.04, 42.71; IR (KBr, cm^{-1}): 3438 (m), 3047(vw), 2956(w), 1573(s), 1452 (s), 1383 (m), 1130(m),788(m), 741 (m). MALDI–TOF–MS: *m/z*, 601.98.

Preparation of [L1-Zn-L1] $[NO_3]_2$ (1): L1 (0.200g, 0.4 mmol) and $Zn(NO_3)_2 \cdot 6H_2O$ (0.06g, 0.2mmol) were dissolved in MeOH (10 mL). The mixture was refluxed for 2 h, then concentrated, filtrated, washed with ethanol, yellow powder was obtained, yield: 76 %. ^1H NMR (d_6-DMSO, 400 MHz) δ (ppm) = 3.14 (d, *J* = 10.1 Hz, 3H), 3.35(m, 2H), 3.63 - 3.45 (m, 2H), 3.90 (dt, *J* = 18.2, 5.1 Hz, 2H), 4.72 (d, *J* = 19.3 Hz, 2H), 7.37 (dt, *J* = 15.1, 7.4 Hz, 1H), 7.65 - 7.48 (m, 2H), 7.77 (dd, *J* = 15.8, 8.3 Hz, 1H), 8.10 - 7.88 (m, 2H), 8.40 - 8.26 (m, 2H), 8.45 (dd, *J* = 23.7, 7.3 Hz, 2H), 8.64 (d, *J* = 7.5 Hz, 1H), 8.94 (d, *J* = 4.6 Hz, 1H), 9.23 - 9.11 (m, 1H), 9.34 – 9.22 (m, 2H), 9.37 (s, 1H), 9.54 (s, 1H). ^{13}C NMR ($CDCl_3$, 150 MHz): δ (ppm) = 158.08, 157.48, 152.10, 145.31, 145.17, 139.90, 137.21, 128.40, 125.14, 124.29, 121.77, 121.45, 119.88, 119.01, 118.16, 118.12, 116.48, 110.83, 109.91, 103.52, 71.77, 70.42, 69.48, 58.62, 54.50; IR (KBr, cm-1): 3414 (s), 2925(w), 2346(w), 1594(m), 1474(w), 1384(s), 1015m), 791 (m); ESI: m/z, cal: 1064.37 found: 532.31. $C_{64}H_{56}ZnN_{10}O_{10}$: Calcd. C 64.56, H 4.74, N 11.76. Found: C 64.64, H 4.78, N 11.72.

Preparation of [L1-Zn-L1] $[PF_6]_2$ (2): L1 (0.200g, 0.4 mmol) and $Zn(OAc)_2 \cdot 2H_2O$ (0.053 g, 0.2 mmol) were dissolved in MeOH (10 mL). The mixture was refluxed for 2 h and then 10mL MeOH containing NH_4PF_6 0.065 g (0.4 mmol) was added in. The mixture stirred for 30 min to give a clear yellow solution. Yellow crystals suitable for X-ray diffraction analysis were obtained after two weeks by slow evaporation of the methanol solution at room temperature. yield: 81 %. ^1H NMR (d_6-DMSO, 400 MHz) δ (ppm) = 3.14 (d, *J* = 10.1 Hz, 3H), 3.33(m, 2H), 3.63 - 3.50 (m, 2H), 3.91 (dt, *J* = 18.2, 5.1 Hz, 2H), 4.70 (d, *J* = 19.3 Hz, 2H), 7.39 (dt, *J* = 15.1Hz, 7.4 Hz, 1H), 7.63 - 7.48 (m, 2H), 7.75 (dd, *J* = 15.8, 8.3 Hz, 1H), 8.10 – 8.00 (m, 2H), 8.40 - 8.28 (m, 2H), 8.44 (dd, *J* = 23.7, 7.3 Hz, 2H), 8.63 (d, *J* = 7.5 Hz, 1H), 8.92 (d, *J* = 4.6 Hz, 1H), 9.23 - 9.10 (m, 1H), 9.32 – 9.22 (m, 2H), 9.36 (s, 1H), 9.55 (s, 1H); ^{13}C NMR ($CDCl_3$, 150 MHz): δ (ppm) = 158.03, 157.45, 152.08, 145.28, 145.12, 139.89, 137.20, 128.35, 125.13, 124.26, 121.74, 121.45, 119,87, 118.94, 118.06, 118.02, 116.38, 110.83, 109.61, 103.32, 71.64, 71.21, 69.81, 59.32, 54.90; IR (KBr, cm^{-1}): 3423 (s), 2915(w), 2513(w), 1796(m), 1472(w), 1118(m), 874(m), 712 (m); EI-MS: *m/z*, *cal:* 1065.37 *found:* 532.67 [M^{2+} 100%]. $C_{64}H_{56}ZnF_{12}N_8P_2O_4$: Calcd. C 56.67, H 4.16, N 8.26. Found: C 56.78, H 3.95, N 8.28.

Preparation of [L2-Zn-L2] $[NO_3]_2$ (3): Complex **3** was prepared by a procedure similar to that of **1** but with L2 (0.241g, 0.4 mmol). yield: 76%. ^1H NMR (d_6-DMSO, 400 MHz) δ (ppm) = 3.14 (d, *J* = 5.1 Hz, 3H), 3.37 - 3.29 (m, 2H), 3.49 (dd, *J* = 11.0, 6.1 Hz, 2H), 4.00 - 3.73 (m, 2H), 4.61 (s, 2H), 7.27 (s, 1H), 7.52 (dd, *J* = 14.2, 7.4 Hz, 4H), 7.70 (dt, *J* = 15.4, 9.1 Hz, 3H), 7.91 - 7.78 (m, 1H), 7.94 (s, 1H), 8.02 (dd, *J* = 11.7, 6.7 Hz, 3H), 8.21 (t, *J* = 10.2 Hz, 1H), 8.32 (t, *J* = 7.7 Hz, 2H), 8.48 (s, 1H), 8.64 – 8.50 (m, 2H), 8.92 (s, 1H), 9.18 (dd, *J* = 33.4, 21.9 Hz, 3H), 9.46 (s, 1H); ^{13}C NMR ($CDCl_3$, 150 MHz): δ (ppm) = 158.06, 157.44, 152.01, 145.13, 141.56, 139.82, 138.03, 137.23, 128.43, 127.32, 126.94, 126.21, 125.96. 125.11, 124.28, 121.74, 121.43, 119.85, 119.71, 118.04, 110.84, 109.81, 109.64, 108.91, 71.45, 71.22, 70.13, 59.34, 54.90; IR (KBr, cm^{-1}): 3434 (s), 2924(w), 2346(w), 1594(w), 1475(w), 1384(s), 1186(m), 1129(m), 1015(w), 691(w); ESI: *m/z*, *cal:* 1268.47, *found:* 634.11; $C_{80}H_{68}ZnN_{10}O_{10}$: Calcd. C 68.89, H 4.91, N 10.04. Found: C 68.75, H 4.97, N 10.08.

Preparation of [L2-Zn-L2] $[PF_6]_2$ (4): Com^{1}H NMR (d_6-DMSO, 400 MHz) δ (ppm) = 3.14 (d, *J* = 5.1 Hz, 3H), 3.37 - 3.28 (m, 2H), 3.48 (dd, *J* = 11.0, 6.1 Hz, 2H), 4.00 - 3.74 (m, 2H), 4.63 (s, 2H), 7.25 (s, 1H), 7.51 (dd, *J* = 14.2, 7.4 Hz, 4H), 7.69 (dt, *J* = 15.4, 9.1 Hz, 3H), 7.91 - 7.77 (m, 1H), 7.93 (s, 1H), 8.02 (dd, *J* = 11.7, 6.7 Hz, 3H), 8.20 (t, *J* = 10.2 Hz, 1H), 8.31 (t, *J* = 7.7 Hz, 2H), 8.47 (s, 1H), 8.62 – 8.50 (m, 2H), 8.91 (s, 1H), 9.19 (d, *J* = 33.4, 21.9 Hz, 3H), 9.45 (s, 1H); ^{13}C NMR ($CDCl_3$, 150 MHz): δ (ppm) = 158.06, 157.44, 152.01, 145.12, 141.56, 139.82, 138.03, 137.23, 128.43, 127.31, 126.94, 126.21, 125.96. 125.11, 124.28, 121.74, 121.43, 119.85, 119.71, 118.04, 110.84, 109.81, 109.64, 108.91, 71.44, 71.22, 70.12, 59.34, 54.91; IR (KBr, cm^{-1}): 3413 (s), 2916(w), 2515(w), 1796(m), 1593(w), 1536(w), 1474(w), 1120(m), 874(m), 712 (m); EI-MS: *m/z*, *cal:* 1270.86, *found:* 635.50 [M^{2+}, 100%]; $C_{80}H_{68}ZnF_{12}N_8P_2O_4$: Calcd. C 61.56, H 4.39, N 7.18. Found: C 62.35, H 4.20, N 7.04.

Materials and Apparatus

All chemicals and solvents were dried and purified by usual methods. Elemental analysis was performed with a Perkin–Elmer 240 analyzer. IR spectra (4000–400 cm–1), as KBr pellets, were recorded on a Nicolet FT–IR 170 SX spectrophotometer. Mass spectra were obtained on a Micromass GCT-MS Spectrometer. ^{1}H and ^{13}C NMR spectra were recorded on a Bruker AV 400 spectrometer with tms as internal standard.

X-ray Crystallography

Single-crystal X-ray diffraction measurements were carried out on a Siemens Smart 1000 CCD diffractometer equipped with a graphite crystal monochromator situated in the incident beam for data collection at room temperature. The determination of unit cell parameters and data collections were performed with Mo-K_α radiation (λ = 0.71069 Å). Unit cell dimensions were obtained with least-squares refinements, and all structures were solved by direct methods with SHELXL-97. All nonhydrogen atoms were located in successive difference Fourier syntheses. The final refinement was performed by full-matrix leastsquares methods with anisotropic thermal parameters for non-hydrogen atoms on F^2. The hydrogen atoms were added theoretically and riding on the concerned atoms. The crystal data and structure refinement for the three compounds are listed in Table S1. Selected bond lengths and angles for the four compounds are summarized in Table S2. The following crystal structures have been deposited at the Cambridge Crystallographic Data Centre and allocated the deposition numbers CCDC **of L2, 2, 4 are 939871, 939806, 939870, respectively.**

Computational studies

To better understand the charge transfer state, density functional theory (DFT) calculations on all the compounds were carried out in vacuo. Optimizations were carried out with B3LYP[LANL2DZ] without any symmetry restraint[3]. The time-dependent density functional theory (TD-DFT) {B3LYP-[LANL2DZ]} calculations, including optimizations and TD-DFT, were implemented with the G03 software[4]. Geometry optimization of singlet-singlet excitation energies were carried out with a basis set composed of 6-31G(d) for C, N, O, F, P and H atoms and the LANL2DZ basis set for Zn atoms. The basis set was downloaded from the EMSL basis set library. The lowest 25 spin-allowed singlet-singlet transitions, up to energy of about 5 eV, were taken into account in the calculation of the absorption spectra.

Optical Measurements

The OPA spectra were measured on a UV-3600 spectrophotometer. The OPEF measurements were performed by using an F-2500 fluorescence spectrophotometer. The concentration of sample solution was 1.0×10^{-5} mol/L. The fluorescence quantum yields (Φ) were determined by using coumarin 307 as the reference according to the literature method[5]. Quantum yields were corrected as follows:

$$\Phi_s = \Phi_r \left(\frac{A_r \eta_s^2 D_s}{A_s \eta_r^2 D_r} \right)$$

Where the *s* and *r* indices designate the sample and reference samples, respectively, *A* is the absorbance at λ_{exc}, η is the average refractive index of the appropriate solution, and *D* is the integrated area under the corrected emission spectrum[6]. For time-resolved fluorescence measurements, the fluorescence signals were collimated and focused onto the entrance slit of a monochromator with the output plane equipped with a photomultiplier tube (HORIBA HuoroMax-4P). The decays were analyzed by 'least-squares'. The quality bof the exponential fits was evaluated by the goodness of fit (χ^2).

Two-Photon Excited Fluorescence (2PEF) Spectroscopy and Two-Photon Absorption (2PA) Cross-Section

TPA cross-sections (δ) of the samples were obtained by the two-photon excited fluorescence (2PEF) method with a femtosecond laser pulse and a Ti:sapphire system (690–860 nm, 80 MHz, 140 fs) as the light source. The concentration of sample solution was 1.0×10^{-3} M. Thus, the δ values of samples were determined by the following Equation (1). $\delta_s=\delta_r \cdot F_s \cdot \Phi_r \cdot C_r \cdot n_r / F_r \cdot \Phi_s \cdot C_s \cdot n_s$ where the subscripts "s" and "r" represent sample and reference (here, fluorescein in ethanol solution at a concentration of 1.0×10^{-3} mol/L was used as reference), respectively. F is the overall fluorescence collection efficiency intensity of the fluorescence signal collected by the fiber spectra meter. Φ, *n* and *c* are the quantum yield of the fluorescence, the refractive index of solvent, and the concentration of the solution, respectively.

There is no linear absorption in the wavelength range 600-900 nm for all the compounds in DMF, which indicates that there are no energy levels corresponding to an electron transition in this spectral range. If frequency upconverted fluorescence appears upon excitation with a tunable laser in this range, it should be safely attributed to multiphoton absorption excited fluorescence. The details of determination conditions are given in the Experimental Section. Detailed experiments revealed that the peak positions of the 2PEF spectra of these chromophores are independent of the excitation wavelengths, but the emission intensities of the TPEF are dependent on the excitation wavelengths. The electrons can be pumped to the different excited states by 2PA due to the different selection rules, but they would finally relax to the same lowest excited state via internal conversion and/or vibrational relaxation[7].

Linear Absorption and One-Photon Excited Fluorescence (1PEF):

Generally, the linear absorption spectra of **L1-L2** feature an intense absorption band in the range of 278–295 nm with shoulder band around 314–406 nm range in different solvents. The low-energy band originating from a HOMO (H)→LUMO (L) transition was assigned to intraligand charge transfer (ICT) transitions, while the high-energy band was assigned to a H-1→L transition (ICT) and mixed

with H-1→L+5 transition due to the π–π* transitions of the tpy. It was further corroborated by calculations (Figure S4). As shown in Figure S3, S4, Table S3, the ICT absorption bands exhibit an weak solvatochromism, indicating there is fairly little difference in dipoles between ground and excited state of the ligand molecule[8]. All the complexes (**1-4)** show intense bands in the range of 281–295 nm, the shoulders around 312-327 nm and broad bands about 370-454 nm region. The bands at 281-295 nm of the complexes are thus contributed from the admixture of the intraligand transitions of tpy. The absorption bands at 312–327 nm with a vibronic structure of 1300 to 1500 cm^{-1} are attributed to the π-π* transitions of the terpyridine ligand[9] (Figure S3). The shoulders around 400 nm are tentatively assigned to MLCT transitions[10]. The influence of the substituents of the ligands on the low-energy band is not profound. Complex **1** results in an obvious bathochromic shift of the π–π*/ICT transition in the linear absorption spectra because the strong Lewis acidity of the metal ion enhances the acceptor strength of the terpyridine moieties[11]. The same shift tendency in emission of the complexes in different solvents was observed for that of **L1** compared to its neutral counterparts (Table S3) which implies that the emitting state could have π–π*/ICT character as well.

Cell Image

Cells (including cancer cells and normal cells) were seeded in 24 glass bottom well plates at a density of $1x10^4$ cells per well and grown for 96 hours. For live cell imaging cell cultures were incubated with the complexes (10% water: 90% cell media) at concentrations 1-10 μM and maintained at 37 °C in an atmosphere of 5% CO_2 and 95% air for incubation times ranging for 0.5 hours. The cells were then washed with PBS (3 x 1 ml per well) and 1 ml of PBS was added to each well. The cells were imaged using confocal laser scanning microscopy and oil immersion lenses at 37 °C in an atmosphere of 5% CO_2 in a ZEISS image chamber. Excitation energy of 820 nm was used and the fluorescence emission measured at 495-582 nm.

Microscopy

For confocal microscopy, cells were luminescently imaged on a Zeiss LSM 710 META upright confocal laser-scanning microscope using magnification 40x, 63x and 100x oil immersion lenses for monolayer cultures, zebrafish and tissue sections. Image data acquisition and processing was performed using Zeiss LSM Image Browser, Zeiss LSM Image Expert and Image J.

For transmission electron microscopy, Cell specimens were received pelleted in Eppendorf tubes. Fresh 3%glutaradehyde in 0.1M phosphate buffer was added to re-suspend the pellet to ensure optimal fixation, and left overnight at 4°C. The specimens were then washed in 0.1M phosphate buffer at 4°C, twice at 30min intervals. Secondary fixation was carried out in 2% aqueous osmium tetroxide for 2 hours at room temperature, followed by washing in buffer as above. Continuing at room temperature,

this was followed by dehydration through a graded series of ethanol: 75% (15min), 95% (15min), 100% (15min) and 100% (15min). 100% ethanol was prepared by drying over anhydrous copper sulphate for 15min. The specimens were then placed in an intermediate solvent, propylene oxide, for two changes of 15mins duration. . Resin infiltration was accomplished by placing the specimens in a 50/50 mixture of propylene oxide/Araldite resin. The specimens were left in this mixture overnight at room temperature. The specimens were left in full strength Araldite resin for 6-8 hrs at room temperature (with change of resin after 3-4 hrs) after which they were embedded in fresh Araldite resin for 48-72 hrs at 60 ^{o}C. Semi-thin sections approximately 0.5 µm thick were cut on a Leica ultramicrotome and stained with 1% Toluidine blue in Borax. Ultra-thin sections, approx. 70-90nm thick, were cut on a Leica ultramicrotome and stained for 25mins with saturated aqueous uranyl acetate followed by staining with Reynold's lead citrate for 5mins. The sections were examined using a FEI Tecnai Transmission Electron Microscope at an accelerating voltage of 80kVv. Electron micrographs were taken using a Gatan digital camera.

Cytotoxicity MTT assays

To ascertain the cytotoxic effect of all the compounds treatment over a 24h period, the 5-dimethylthiazol-2-yl-2,5-diphenyltetrazolium bromide (MTT) assay was performed. HepG2 cells were trypsinized and plated to ~70% confluence in 96-well plates 24 h before treatment. Prior to the compounds' treatment, the DMEM was removed and replaced with fresh DMEM, and aliquots of the compound stock solutions (500 µ in water) were added to obtain final concentrations of 20, 40, 60, 80 and 100 µM. The treated cells were incubated for 24 h at 37 ^{o}C and under 5% CO_2. Subsequently, the cells were treated with 5 mg/mL MTT (40 µL/well) and incubated for an additional 4 h (37 ^{o}C, 5% CO_2). Then, DMEM was removed, the formazan crystals were dissolved in DMSO (150 µL/well), and the absorbance at 490 nm was recorded. The cell viability (%) was calculated according to the following equation: cell viability % = OD_{490}(sample)/OD_{490}(control) ×100, where OD_{490}(sample) represents the optical density of the wells treated with various concentration of the compounds and OD_{490}(control) represents that of the wells treated with DMEM + 10% FCS. Three independent trials were conducted, and the averages and standard deviations are reported. The reported percent cell survival values are relative to untreated control cells.

MCs formation

In order to produce MCs, a layer of poly(2-hydroxyethyl methacrylate) (polyHEMA) thin film was coated on the bottom of tissue culture flasks. To ensure sterile, polyHEMA coated flask must be exposed to ultraviolet light for 2 h before use. HepG2 monolayer cells incubated as mentioned above were trypsinized to give a single-cell suspension and count the cell numbers using a hemocytometer. 5×10^5 cells in 5 mL of fresh DMEM medium was placed in a cell culture flask coated by polyHEMA. Cells were incubated at 37 ^oC in humidified atmosphere with 5% CO_2 and the culture medium was replaced every other day. HepG2 MCs (around 300 μm in diameter) formed spontaneously in 7 days.

Animals: All procedures involving animals were approved by and conformed to the guidelines of the Anhui University Animal Care Committee, School of life science. We have taken great efforts to reduce the number of animal used in these studies and also taken effort to reduce animal suffering from pain and discomfort.

In vivo assessment of 1 in zebrafish model

Either zebrafish larva and adult were purchased from the Anhui University Zebrafish Breed House, School of life science. Larval and adult zebrafish were then soaking in 1μM of **1** for the time that the experiment required. All the fish were terminally anaesthetized using MS222 at class III (Deep Anaesthetized), followed by microscopy, co-staining experiment or sectioning.

In vivo assessment of 1 in mouse model

3 mouth old SPF (Specific pathogen Free) Kunming mouse were intravenously (i.v.) injected via the tail vein with 10μM **1**, the volume of solution injected was 8% of the total blood volume (TBV). TBV was calculated as 58.5 mL of blood per kg of body weight. The mouse received 2 injections within 24 hours (interval=12 hours, t_1=0 hour, t_2=12th hour). 5 min before sacrificing, mice were initially injected (i.v.) with 200μL of fluorescein-labelled lectins (FL-1174 Vector Labs, UK) at 0.5 mg/mL to label the microvasculature in vivo. The lectin solution was allowed to circulate for 2 min, following which the mice were terminally anaesthetised and transcardially perfused with phosphate buffered saline (PBS) 0.1 M pH 7.4. Their brains, liver and kindney were extracted and then snap frozen in liquid nitrogen cooled isopentane. Fresh tissues from PBS-perfused animals were sectioned at 20μm in the sagittal plane using a cryostat (Leica 1950). Sections were mounted on glass slides, the nuclei stained with 4’,6-diamidino-2-phenylindole dilactate (DAPI; 500 nM in PBS) for 1 min and cover-slipped using an aqueous mountant (Vectashield, Vector Labs).

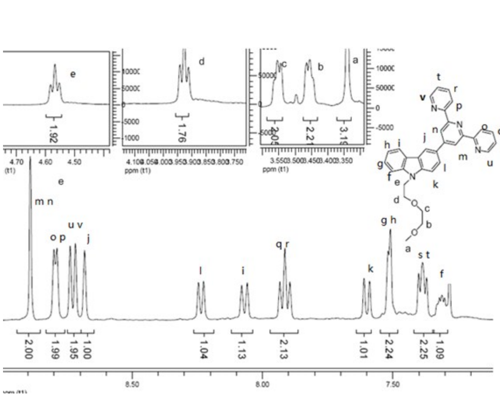

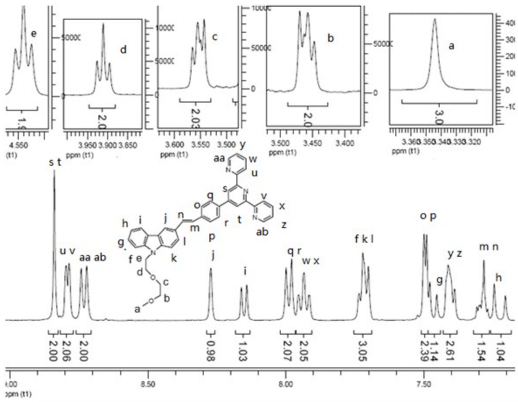

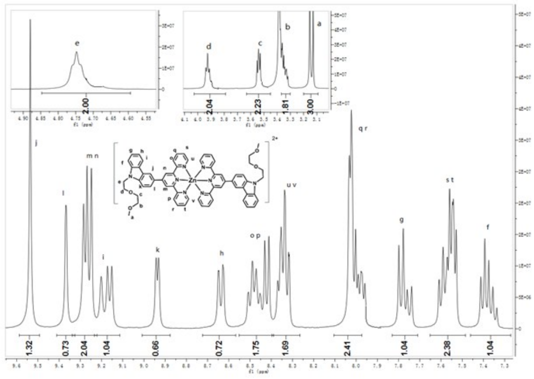

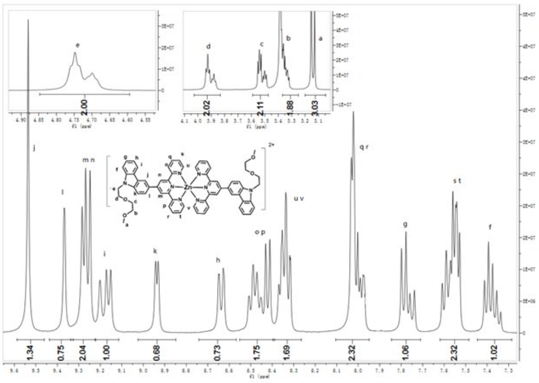

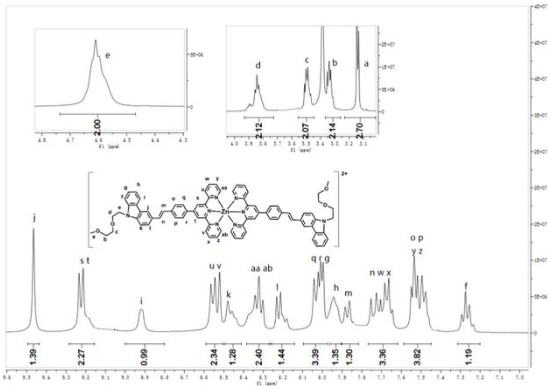

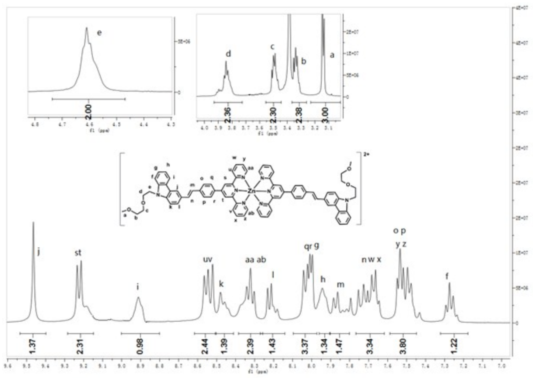

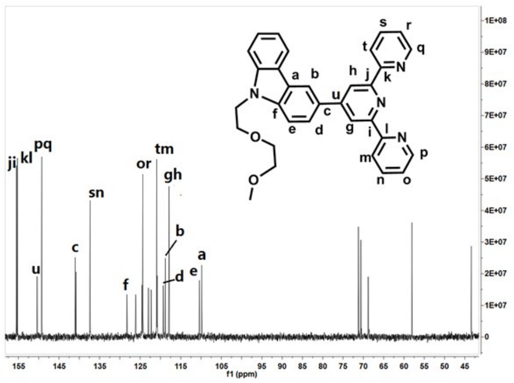
f1 (ppm)

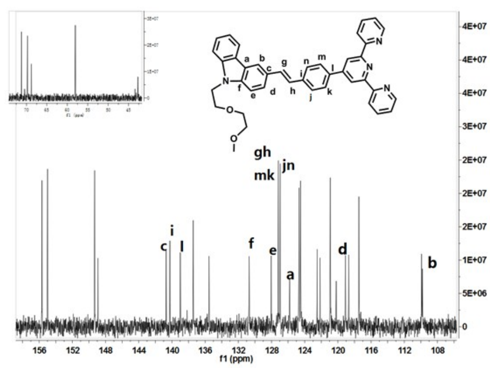
f1 (ppm)

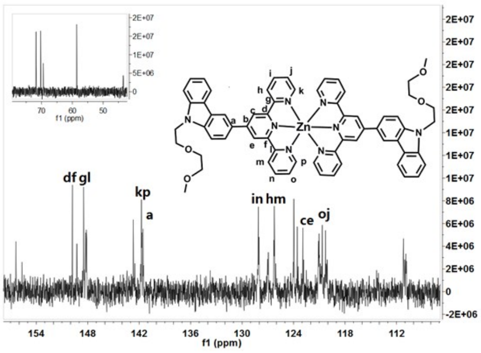
df gl
kp
a
in hm
ce
oj
Zn
f1 (ppm)

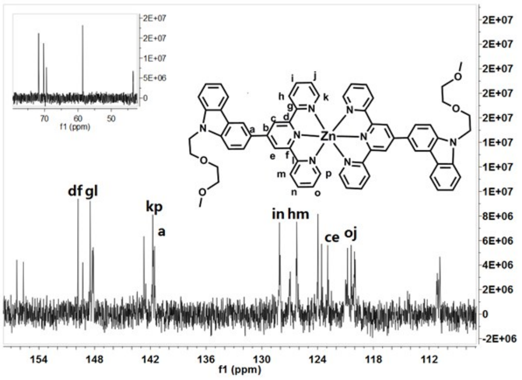
df gl
kp
a
in hm
ce
oj
Zn
f1 (ppm)

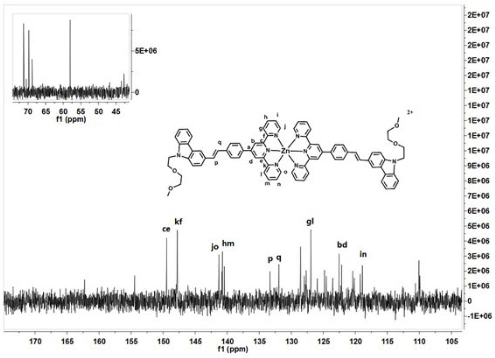
ce
kf
jo
hm
p
q
gl
bd
in
f1 (ppm)

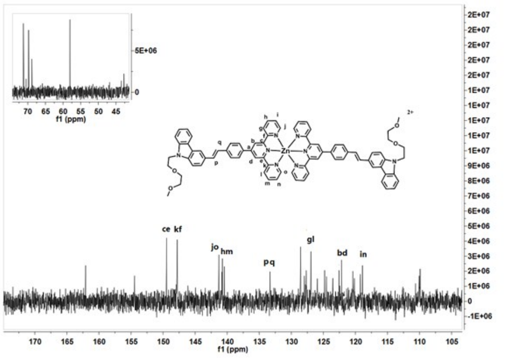
ce
kf
jo
hm
pq
gl
bd
in
f1 (ppm)

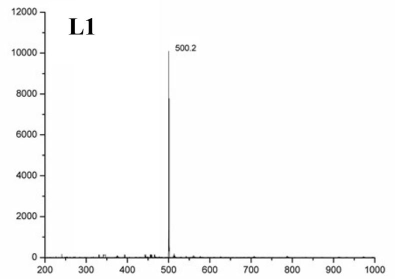
L1
500.2

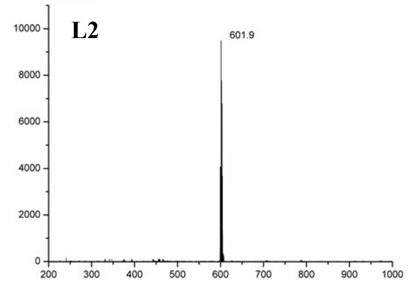
L2
601.9

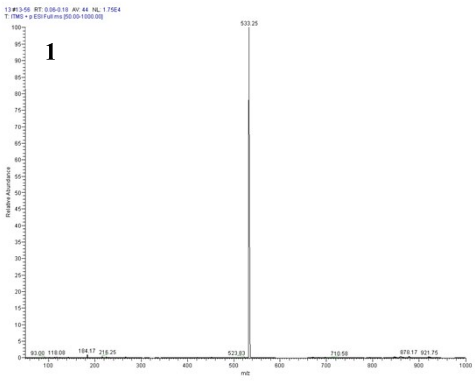
1
533.25

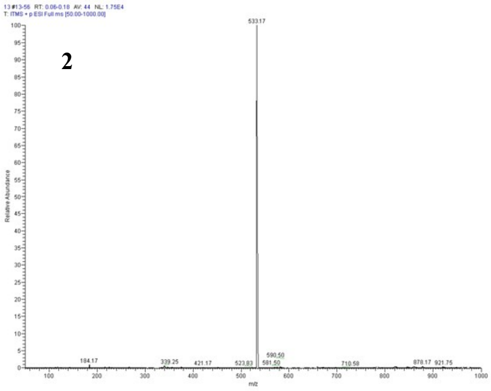
2
533.17

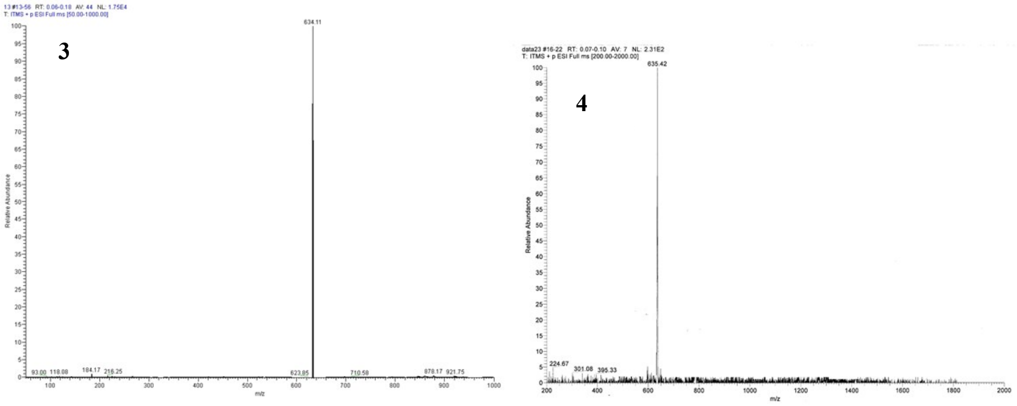

Figure S1. ^{1}H NMR ^{13}C NMR and MS spectra of all the compounds.

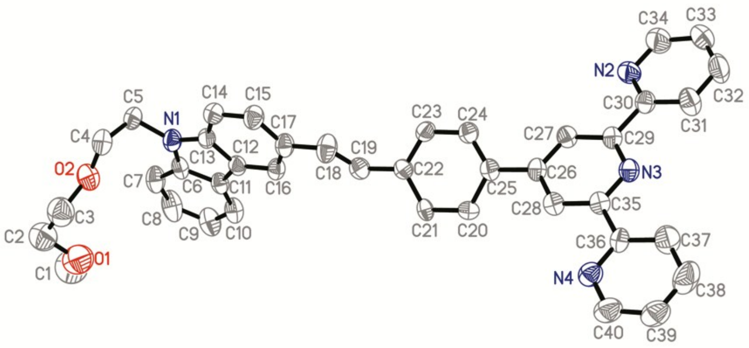

Figure S2. The molecular structures of the ligands **L2**.

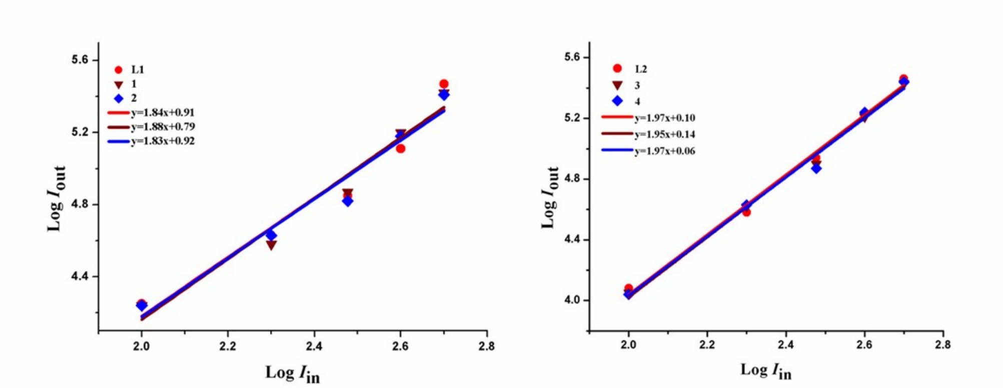

Figure S3. Log–Log linear of squared dependence of induced fluorescence signal and incident irradiance intensity of two ligands and complexes **L1** to **L2** and **1-4**.

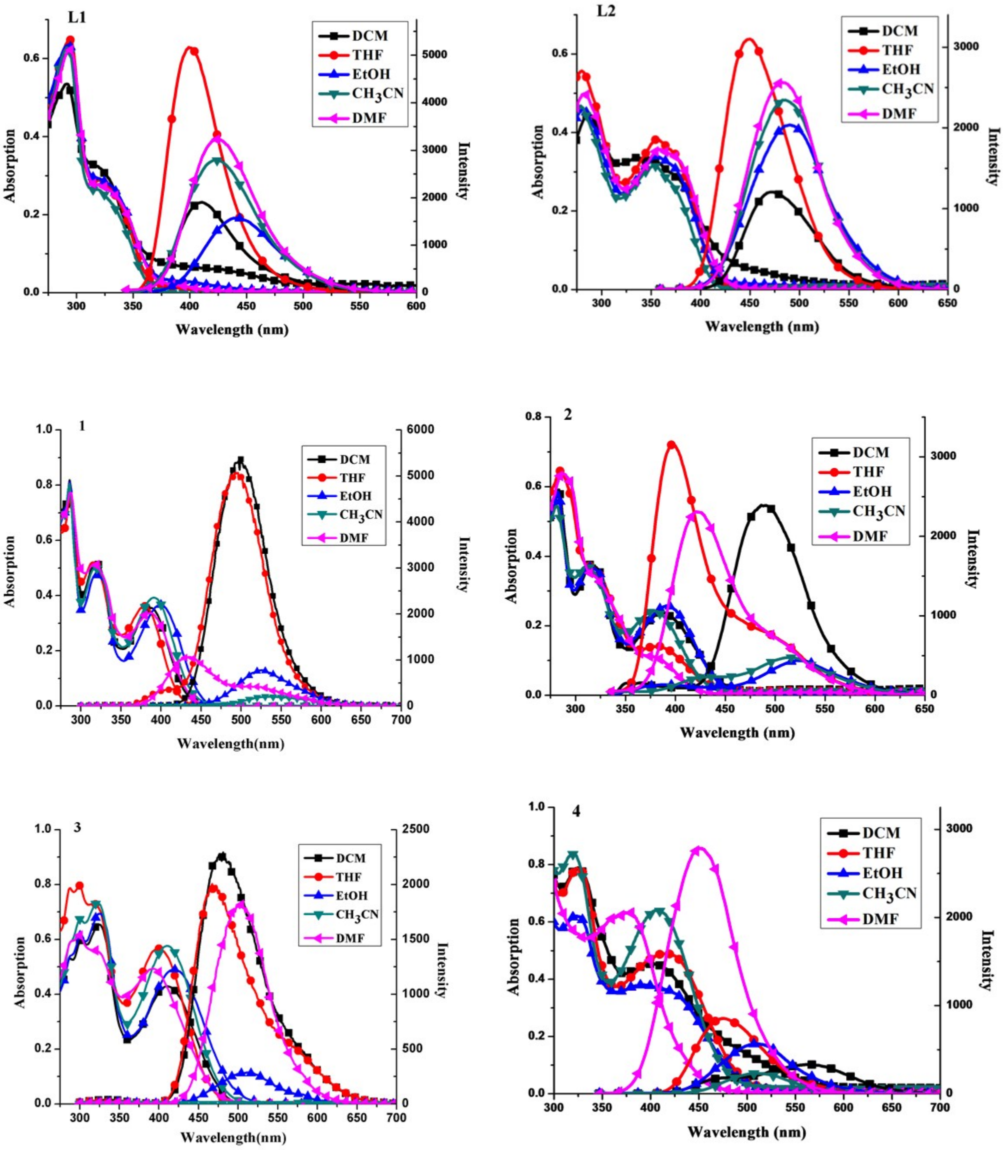

Figure S4. Linear absorption and linear emission spectra of **L1, L2, 2, 3** and **4** in five organic solvents with a concentration of 1×10^{-5} mol/L.

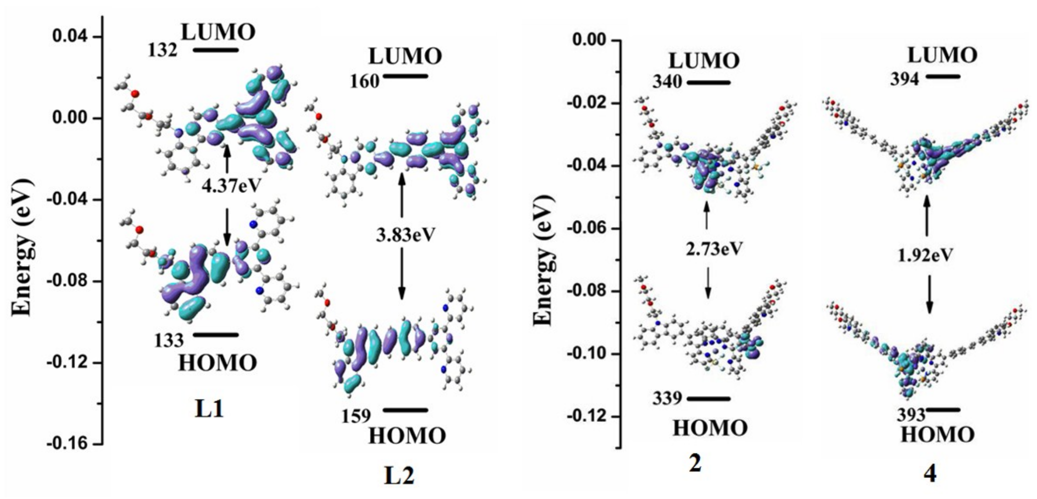

Figure. S5 Representation of calculated Kohn-Sham orbitals of **L1, L2, 2** and **4**

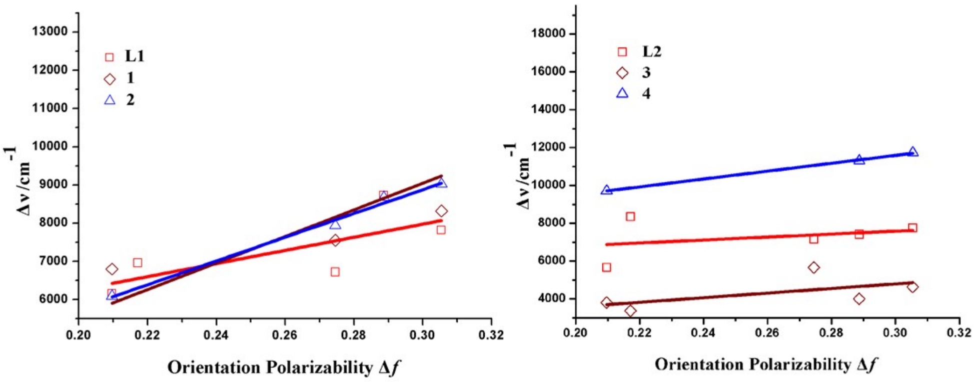

Figure. S6 Lippert-Mataga plots for all the compounds.

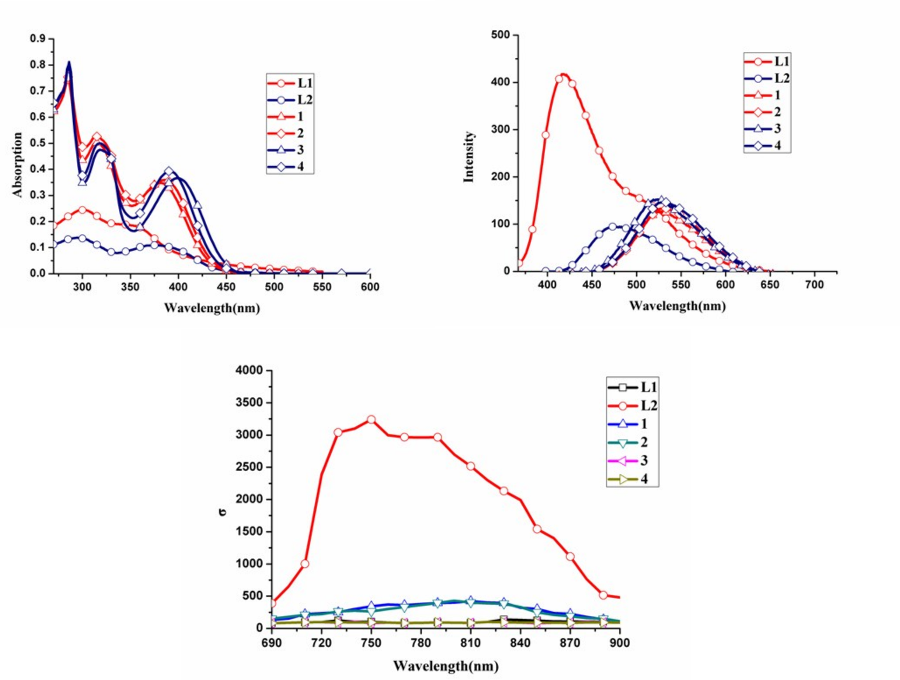

Figure S7. Linear absorption, linear emission(1×10^{-5} mol/L) and TPA (1×10^{-3} mol/L) spectra of L1, L2, 1, 2, 3 and 4 in DMSO:H2O=1:9 solution.

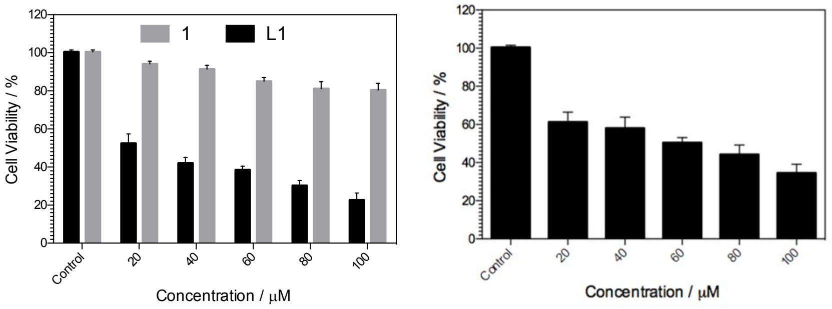

Figure S8. Cytotoxicity data of **1, L1** (left) and **L2** (right) obtained from the MTT assay.

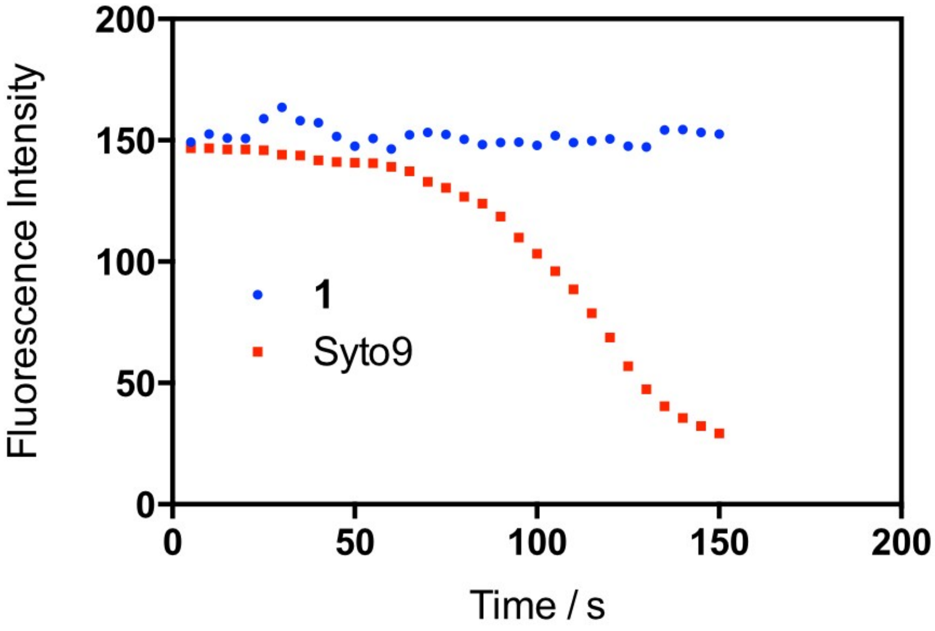

Figure S9. Photon bleaching test of **1** and Syto-9 in living cells under continues laser irradiation.

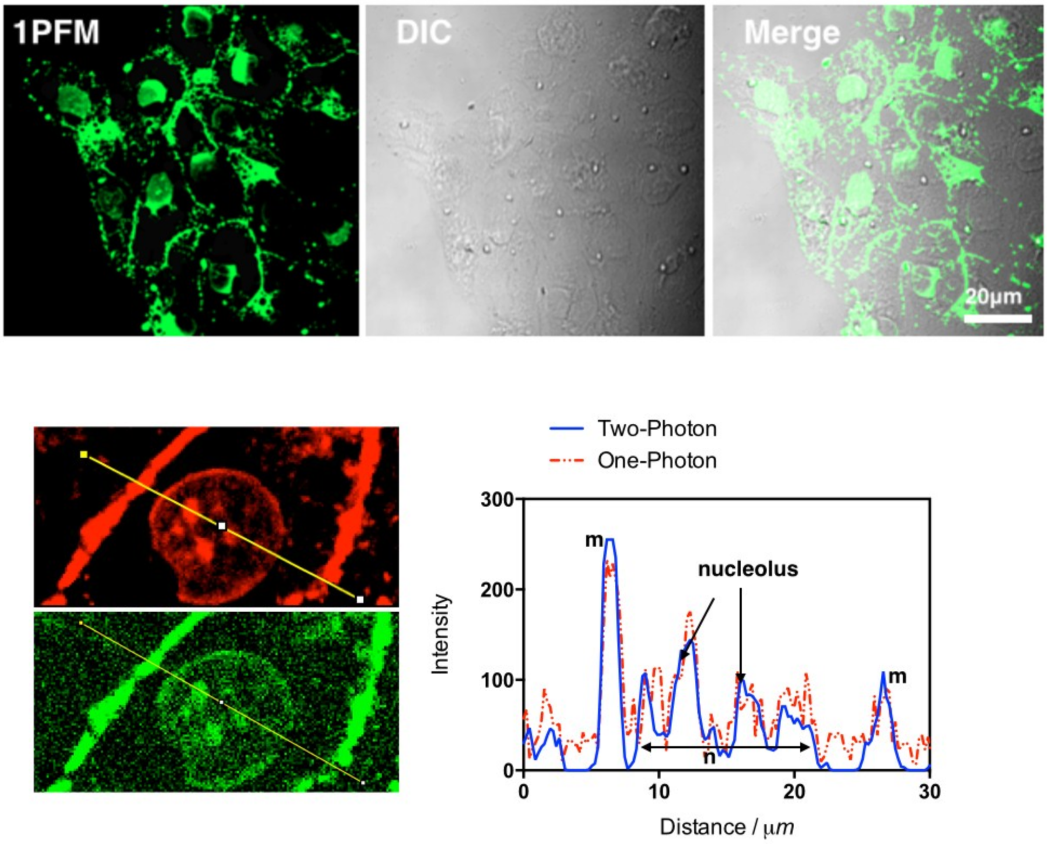

Figure S10. One-photon (green) micrographs for living cells treated with **1**; Two-photon (red) and One-photon (green) micrographs for living cells treated with **1**, and single cell fluorescence intensity profile. m=membrane, n=nucleus.

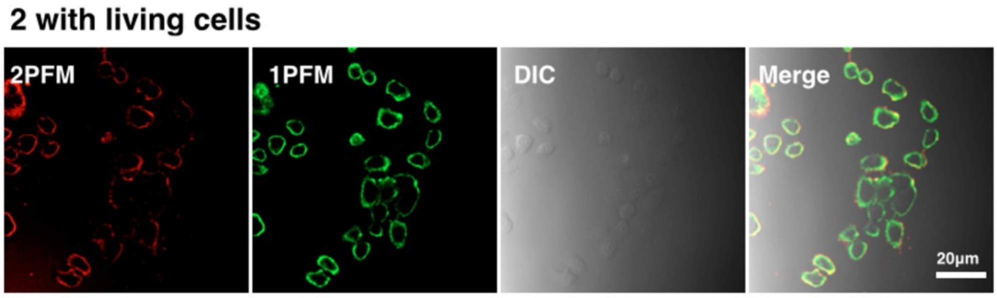

Figure S11. Two-photon (red) and one-photon (green) micrographs for living cells treated with complex **2**.

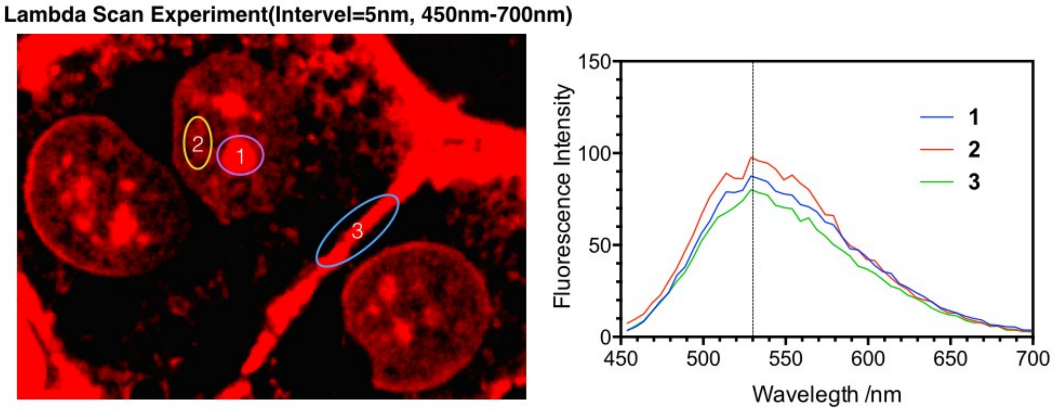

Figure S12 Lambda-scanning experiment performed for cells treated with **1** and ROI (Region of Interesting) intensity analysis (interval=5nm).

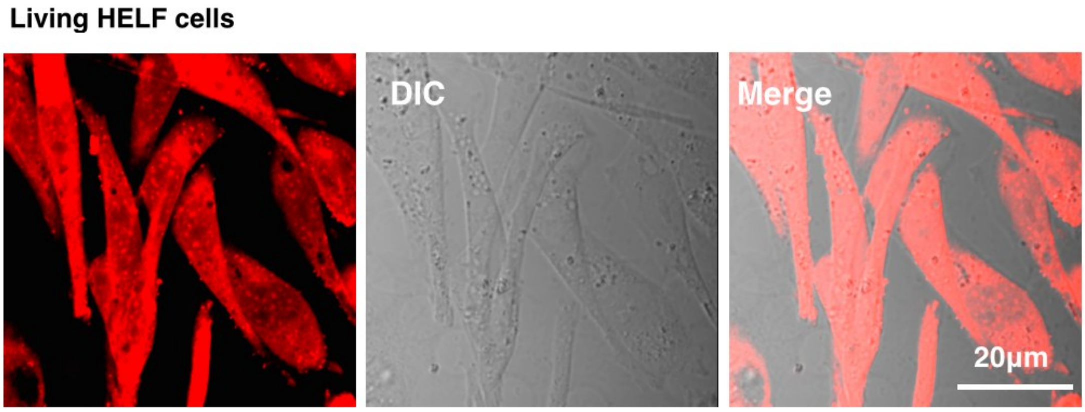

m.

Figure S13. Two-photon micrographs for living HELF cells treated with **1** and merged with DIC channel.

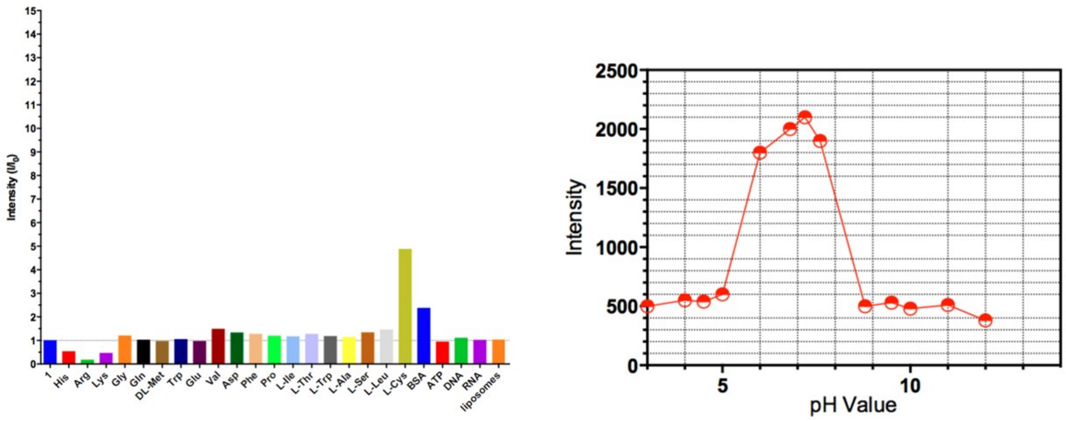

Figure S14. Fluorescence intensity measurement using **1** with different substances (left); Fluorescence intensity measurement using **1** at different pH (right).

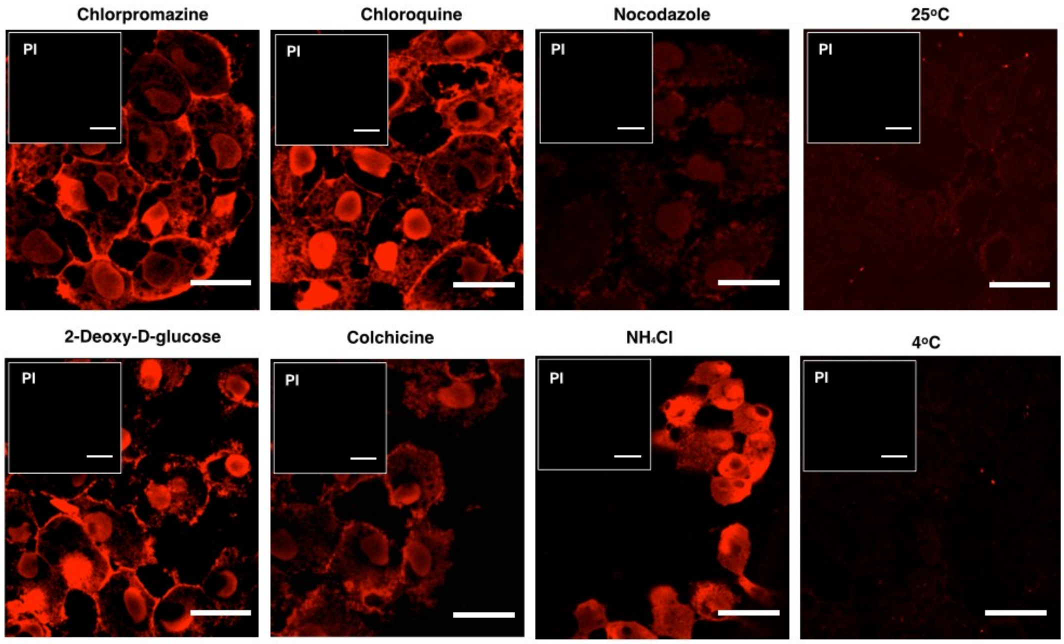

Figure S15. Two-photon micrographs for living HepG2 cells treated with **1** and different inhibitors, subsequently co-stained with PI to indicated the cell mortality. The scale bars represent 20μm.

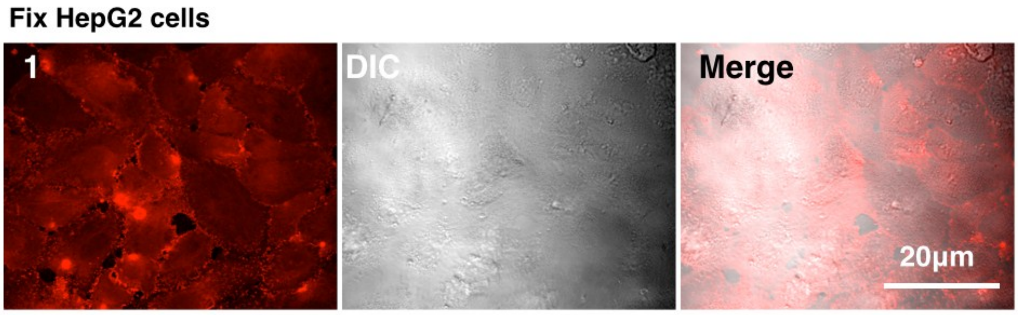

Figure S16. Two-photon micrographs for pre-fixed HepG2 cells treated with **1**.

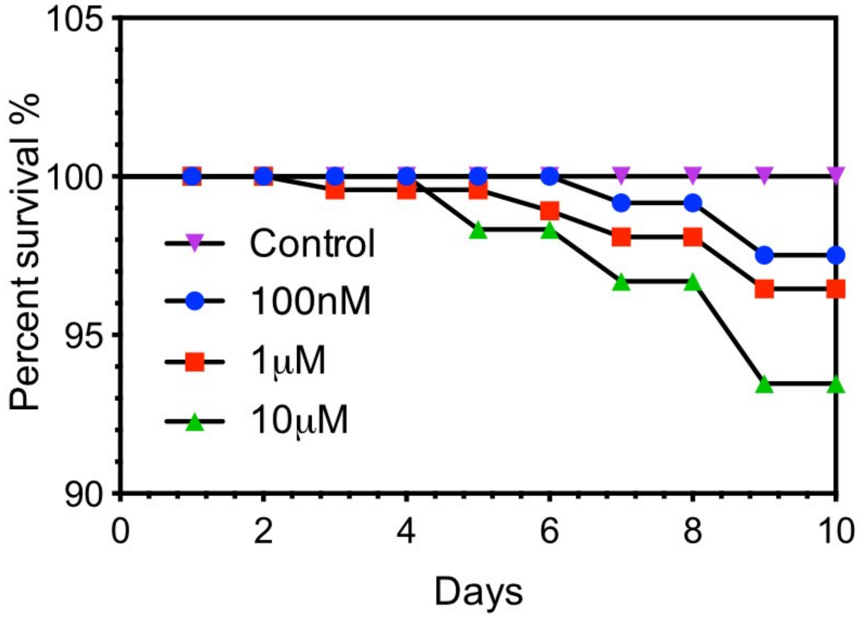

Figure S17. Survival rate of larval zebrafish treated with **1** for up to 10 days.

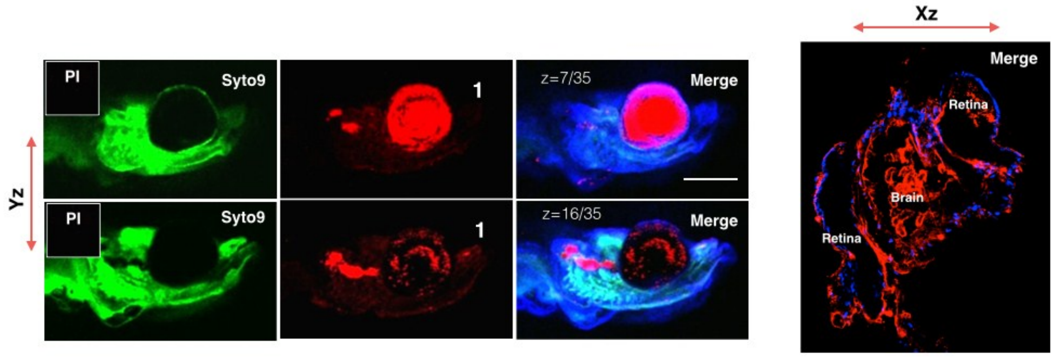

Figure S18. 2D Yz sections (Z=7/35 and 16/35) and Xz sections from Figure 3a of staining larval zebrafish by **1**.

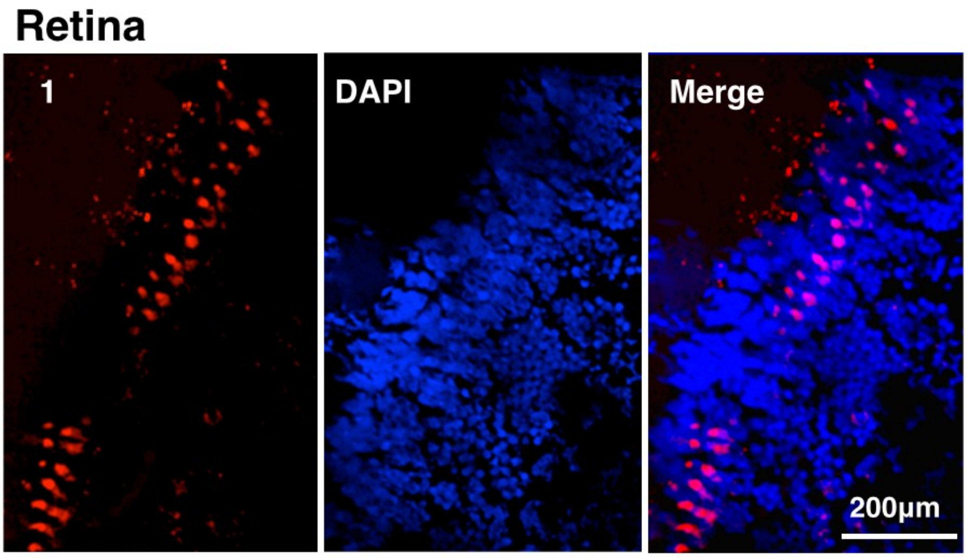

Figure S19. Retina section of adult zebrafish treated with **1**, imaged under 2PFM.

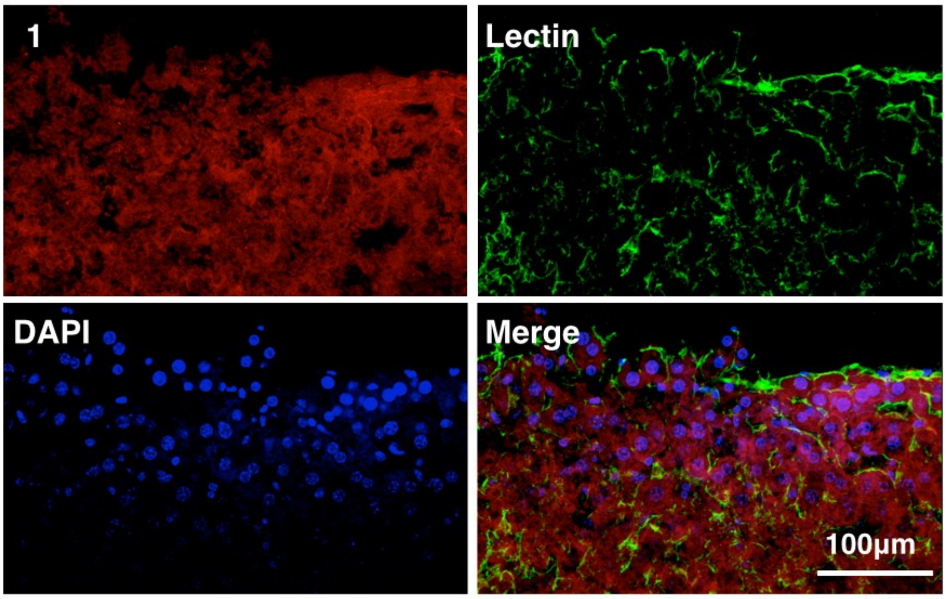

Figure S20. Liver section of mouse IV administrated with **1**, co-stained with Lectin and DAPI, imaged under 2PFM

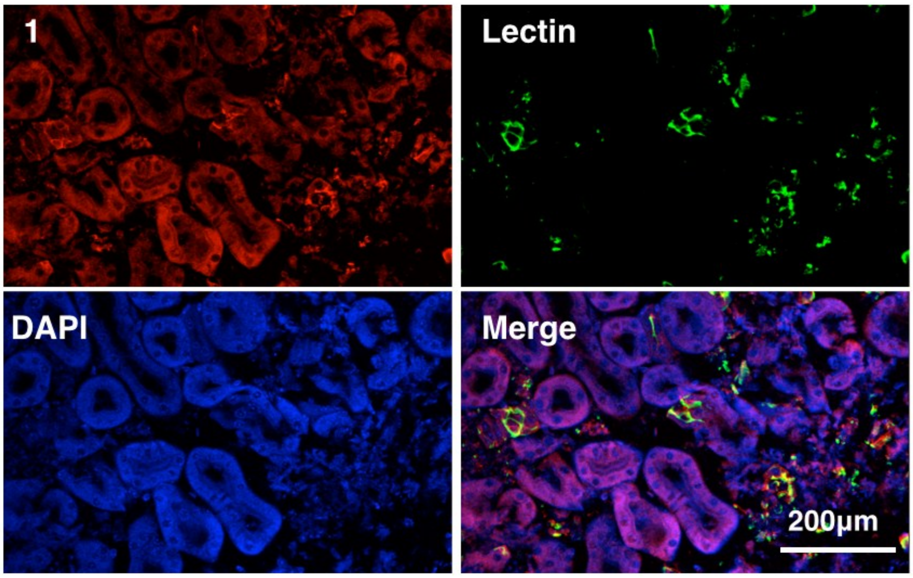

Figure S21. Kidney section of mouse IV administrated with **1**, co-stained with Lectin and DAPI, imaged under 2PFM

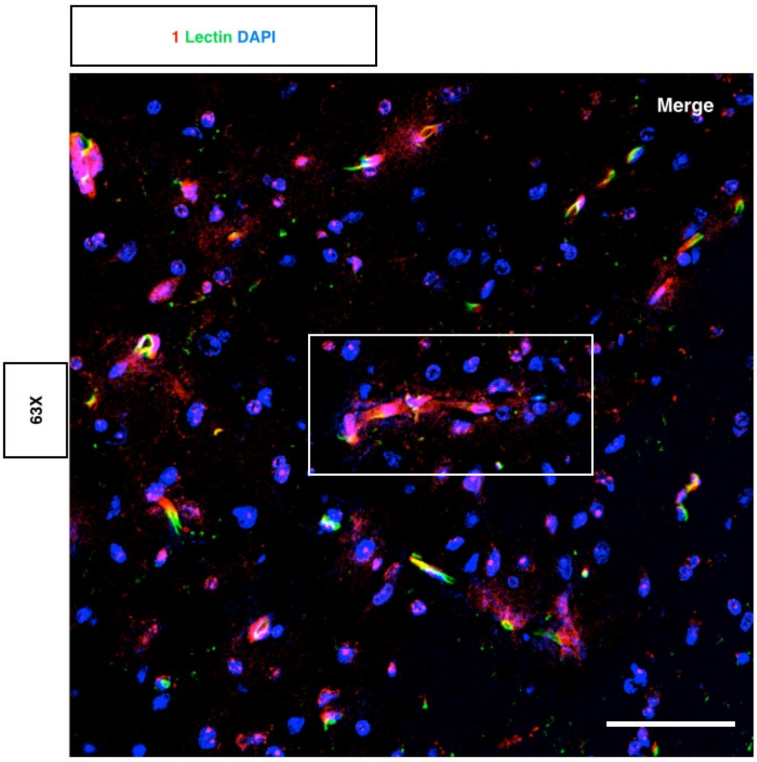

Figure S22. Higher resolution (63X) micrographs show the brain distribution of **1**.

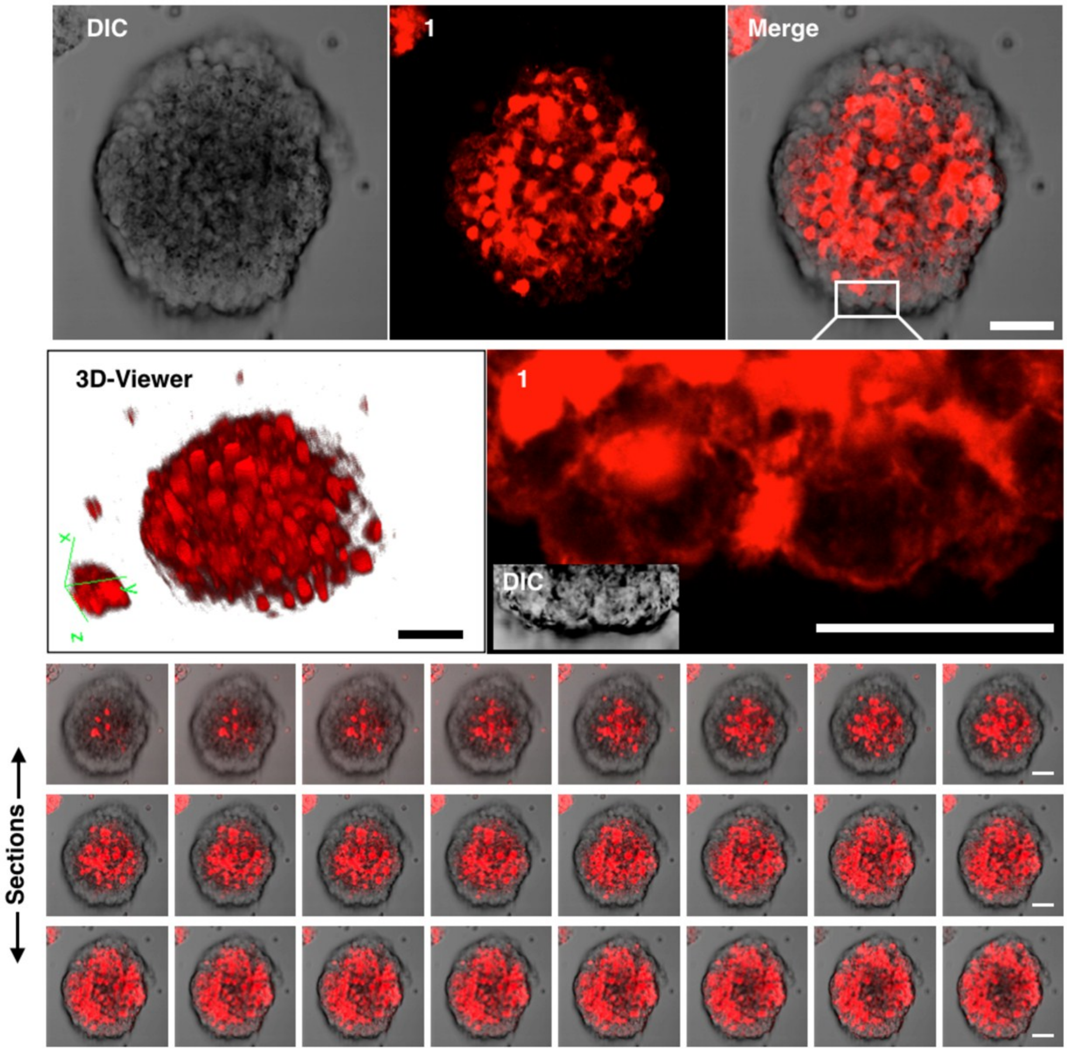

Figure S22. Complex **1** (1μM, 3 hours incubation) diffusion capability using HepG2 multi-cells spherical (MCs) as a solid tumor model, imaged under confocal laser scanning microscopy, the scale bar represented 100μm.

Table S1. Crystallographic data and structure refinement for **L2**, **2 and 4**.

Compound	**L2**	**2**	**4**
CCDC	939871	939806	939870
formula	$C_{40} H_{34} N_4 O_2$	$C_{64} H_{34} F_6 N_8 O_4 P Zn$	$C_{85}H_{75.50}F_{12} N_{10.50}O_4 P_2 Zn$
Formula weight	602.71	1189.33	1663.37
T/K	293(2)	291(2)	298(2)
$\lambda_{(Mo-K\alpha)}$ Å	0.71069	0.71069	0.71069
crystal system	Triclinic	monoclinic	Triclinic
space group	P$\bar{1}$	C2/c	P$\bar{1}$
a/Å	5.559(5)	19.019(5)	13.732(5)
b/Å	11.240(5)	21.295(5)	15.892(5)
c/Å	13.638(5)	33.784(5)	19.260(5)
α/deg	75.767(5)	90.000(5)	103.178(5)
ß /deg	83.930(5)	96.721(5)	90.598(5)

γ/deg	81.445(5)	90.000(5)	91.070(5)
V/Å^3	814.7(9)	13589(5)	4091(2)
Dc/Mg m^{-3}	1.229	1.163	1.350
Z	1	8	2
F(000)	318	4840	1718
μ/mm^{-1}	0.077	0.449	0.423
reflections collected	5949	47946	29661
reflections unique	4575	11937	14799
R(int)	0.0296	0.0309	0.0592
data/restraints/params	4575/148/ 91	11937/884/668	14799/0/1050
Final R indices[I>2σ(I)]	R_1=0.0661,wR_2=0.1570	R_1= 0.0651, wR_2=0.2144	R_1=0.0847 wR_2=0.2587
GOF on F^2	1.042	1.085	1.005

Table S2. Selected intra- and intermolecular bond lengths [Å] and angles [°] for **L2**, **2 and 4**.

		**L2**			
C(1)-O(1)	1.449(5)	O(1)-C(2)	1.450(5)	C(3)-O(2)	1.450(5)
C(4)-O(2)	1.421(6)	C(5)-N(1)	1.463(6)	C(6)-N(1)	1.395(6)
C(13)-N(1)	1.378(6)	C(30)-N(2)	1.331(6)	C(34)-N(2)	1.376(7)
C(1)-O(1)-C(2)	109.9(19)	O(1)-C(2)-C(3)	104.7(17)	O(2)-C(3)-C(2)	119.1(16)
N(1)-C(5)-C(4)	111.6(4)	C(7)-C(6)-N(1)	129.4(5)	N(1)-C(6)-C(11)	108.4(4)
N(1)-C(13)-C(14)	129.1(5)	N(1)-C(13)-C(12)	109.0(4)	N(3)-C(29)-C(27)	122.9(4)
N(3)-C(29)-C(30)	116.1(4)	N(2)-C(30)-C(31)	122.9(5)	N(2)-C(30)-C(29)	116.2(4)
C(33)-C(34)-N(2)	124.1(6)	N(4)-C(36)-C(37)	121.1(5)	N(4)-C(36)-C(35)	117.8(4)
		**2**			
Zn(1)-N(1)	2.201(3)	Zn(1)-N(3)	2.177(3)	Zn(1)-N(6)	2.054(3)
Zn(1)-N(2)	2.062(3)	Zn(1)-N(5)	2.185(3)	Zn(1)-N(7)	2.199(3)
N(2)-Zn(1)-N(6)	176.90(10)	N(6)-Zn(1)-N(3)	106.87(10)	N(2)-Zn(1)-N(3)	76.18(10)
N(6)-Zn(1)-N(5)	75.47(10)	N(2)-Zn(1)-N(5)	105.19(10)	N(3)-Zn(1)-N(5)	92.61(10)
N(6)-Zn(1)-N(7)	76.22(10)	N(2)-Zn(1)-N(7)	103.20(10)	N(3)-Zn(1)-N(7)	93.34(11)
N(5)-Zn(1)-N(7)	151.60(11)	N(6)-Zn(1)-N(1)	101.53(11)	N(2)-Zn(1)-N(1)	75.40(11)
		**4**			
Zn(1)-N(6)	2.066(4)	Zn(1)-N(3)	2.078(4)	Zn(1)-N(4)	2.166(4)
Zn(1)-N(5)	2.172(4)	Zn(1)-N(7)	2.183(4)	Zn(1)-N(2)	2.198(5)
C(14)-O(1)	1.417(10)	O(3)-C(78)	1.448(15)	O(3)-C(79)	1.653(16)
O(4)-C(16)	1.40(3)	O(4)-C(83)	1.66(3)	C(86)-N(11)	1.31(2)
N(6)-Zn(1)-N(3)	171.97(16)	N(6)-Zn(1)-N(4)	111.16(16)	N(3)-Zn(1)-N(4)	75.32(16)
N(6)-Zn(1)-N(5)	75.62(16)	N(3)-Zn(1)-N(5)	109.09(16)	N(4)-Zn(1)-N(5)	95.22(16)
N(6)-Zn(1)-N(7)	75.56(16)	N(3)-Zn(1)-N(7)	100.20(16)	N(4)-Zn(1)-N(7)	89.88(16)

Table S3 Photophysical data of all the compounds

	Solvents	λ_{max}[a]	ε[b]	λ_{max}[c]	Φ[d]	λ_{max}[e]	λ_{max}[f]	δ[g]	τ[h]	Δν[i]	Δμ[j]
**L1**	DCM	290<br>319	5.32<br>3.22	410	0.16				2.69	6958	
	THF	294<br>321	6.54<br>2.83	400	0.31				2.57	6153	
	EtOH	291<br>319	6.34<br>2.95	442	0.13				2.61	8724	18.7
	CH_3CN	292<br>319	6.28<br>2.62	425	0.23				2.40	7819	
	DMF	294<br>330	6.18<br>2.66	424	0.26	540	700	107	4.64	6718	
**L2**	DCM	290<br>339	4.42<br>3.40	473	0.15				1.39	8357	
	THF	280<br>358	5.57<br>3.78	449	0.28				1.37	5661	
	EtOH	280<br>360	4.54<br>3.35	491	0.28				1.91	7411	14.28
	CH_3CN	278<br>352	4.64<br>3.19	484	0.29				1.84	7748	
	DMF	280<br>359	4.97<br>3.57	483	0.29	558	730	677	1.85	7151	
**1**	DCM	283<br>318<br>384<br>454	7.54<br>5.10<br>2.62<br>1.90	489	0.17				3.71	10997	
	THF	284<br>319<br>398	8.66<br>5.23<br>3.50	393<br>508	0.11				2.54	5903<br>11762	
	EtOH	284<br>320<br>404	9.27<br>5.46<br>4.11	459<br>531	0.05				2.89	9464<br>12418	40.4
	CH_3CN	282<br>315<br>384	8.42<br>4.95<br>2.34	437<br>509	0.12				2.77	8863<br>12099	
	DMF	288<br>320<br>384	8.69<br>4.79<br>1.57	425	0.19	543	810	234	4.69	7721	
**2**	DCM	283<br>314<br>388	5.84<br>3.77<br>2.36	486	0.24				3.91	11271	
	THF	283<br>312<br>385	6.49<br>3.83<br>1.44	396	0.26				2.41	6799	
	EtOH	282<br>315<br>395	5.79<br>3.72<br>2.61	528	0.06				2.66	12807	44.9
	CH_3CN	281<br>314<br>377	5.38<br>3.72<br>2.42	425<br>516	0.07				2.48	8318<br>12467	
	DMF	287<br>320<br>384	6.36<br>3.42<br>1.06	422	0.21	537	750	245	4.63	7553	
**3**	DCM	302<br>324<br>410	6.03<br>6.55<br>4.30	481	0.24				1.42	3600	
	THF	300<br>400	7.96<br>5.66	469	0.17				1.45	3678	35.32

			6.13						1.79	4240	
		302	6.94								
		324	4.90	511	0.03						
		420									
	EtOH										
			6.75						1.05		
		300	7.34							-	
		322	5.77	-	-						
		410									
	CH_3CN										
			6.15			545	740	159	1.82	7161	
		300	4.94	504	0.17						
		392									
	DMF										
		298	7.63						1.38	13225	
**4**		325	7.88	570	0.04						
	DCM	403	4.55								
		300	7.30						1.43	9717	
		325	7.91	475	0.06						
	THF	419	4.89								
		294	6.08						1.76	11313	
		323	6.21	509	0.06						36.86
	EtOH	403	3.73								
		295	7.81						1.02	11740	
		319	8.35	510	0.02						
	CH_3CN	407	6.45								
		286	8.01	450	0.19	544	700	143	1.78	4374	
	DMF	376	6.35								

[a] Absorption peak position in nm (1×10^{-5} mol L^{-1}). [b] Maximum molar absorbance in 10^4 mol^{-1} L cm^{-1}. [c] Peak position of SPEF in nm (1.0×10^{-5} mol L^{-1}), excited at the absorption maximum. [d] Quantun yields determined by using coumarin 307 (Φ = 0.56) (1.0×10^{-5} mol L^{-1}) as the standard. [e] TPEF peak position in nm pumped by femtosecond laser pulses at 300 mw at their maximum excitation wavelength. [f] TPA maximum excitation wavelength. [g] 2PA cross section in GM. [h] The fitted fluorescence lifetime in ns. [h] fluorescence liftime (ns). [i] Stokes shift in cm^{-1}. [j] $\Delta\mu$ the dipole moment changes of the compounds with photoexcitation (1D=3.334×10^{-30} c·m).

Table S4. Measured values for ϕ_{QEff} for L1 and 1.

compound	DCM	THF	Ethanol	Acetonitrile	DMF
L1	0.84	0.69	0.87	0.77	0.74
1	0.76	0.74	0.94	0.93	0.79

Table S5. Calculated two-photon-related photophysical properties of L1 and L2 in gas phase

MOL	E_{max}	λ^{tp}_{max}	σ_{tp}
L1	3.69	670.22	0.00
	3.78	654.26	145.55
	4.06	609.14	1.01
	4.10	603.20	0.00
L2	3.40	727.38	751.48
	3.52	702.59	0.20
	3.87	639.04	13.72
	3.98	621.38	58.39

[1] W. Yang, Y. Wong, O. T. Ng, L. P. Bai, D. W. Kwong, Y. Ke, Z. H. Jiang, H. W. Li, K. K. Yung, M. S. Wong, *Angew. Chem. In. Ed.* **2012**, *51*, 1804.

[2] Z.-J. Hu, J.-X. Yang, Y.-P. Tian, X.-T. Tao, L. Tian, H.-P. Zhou, G.-B. Xu, W.-T. Yu, Y.-X. Yan, Y.-H. Sun, *B. Chem. Soc. JPN.* **2007**, *80*, 986.

[3] a) A. R. Cowley, J. Davis, J. R. Dilworth, P. S. Donnelly, R. Dobson, A. Nightingale, J. M. Peach, B. Shore, D. Kerr, L. Seymour, *Chem. Commun.* **2005**, *845*; b) J. P. Holland, F. I. Aigbirhio, H. M. Betts, P. D. Bonnitcha, P. Burke, M. Christlieb, G. C. Churchill, A. R. Cowley, J. R. Dilworth, P. S. Donnelly, *Inorg. Chem.* **2007**, *46*, 465; c) J. P. Holland, P. J. Barnard, S. R. Bayly, H. M. Betts, G. C. Churchill, J. R. Dilworth, R. Edge, J. C. Green, R. Hueting, *Eur. J. Inorg. Chem.* **2008**, *2008*, 1985; d) S. I. Pascu, P. A. Waghorn, T. D. Conry, H. M. Betts, J. R. Dilworth, G. C. Churchill, T. Pokrovska, M. Christlieb, F. I. Aigbirhio, J. E. Warren, *Dalton Trans.* **2007**, 4988; e) S. I. Pascu, P. A. Waghorn, T. D. Conry, B. Lin, H. M. Betts, J. R. Dilworth, R. B. Sim, G. C. Churchill, F. I. Aigbirhio, J. E. Warren, *Dalton Trans.* **2008**, 2107.

[4] Z. Ji, Y. Li, T. M. Pritchett, N. S. Makarov, J. E. Haley, Z. Li, M. Drobizhev, A. Rebane, W. Sun, *Chem. Eur. J.* **2011**, *17*, 2479.

[5] Q. Zheng, G. S. He, P. N. Prasad, *J. Mater. Chem.* **2005**, *15*, 579.

[6] G. A. Crosby, J. N. Demas, *J. Phys. Chem.* **1971**, *75*, 991.

[7] T. G. Gray, C. M. Rudzinski, E. E. Meyer, R. Holm, D. G. Nocera, *J. Am. Chem. Soc.* **2003**, *125*, 4755.

[8] H. Y. Woo, B. Liu, B. Kohler, D. Korystov, A. Mikhailovsky, G. C. Bazan, *J. Am. Chem. Soc.* **2005**, *127*, 14721.

[9] a) V. W.-W. Yam, R. P.-L. Tang, K. M.-C. Wong, K.-K. Cheung, *Organometallics* **2001**, *20*, 4476; b) V. W.-W. Yam, R. P.-L. Tang, K. M.-C. Wong,

C.-C. Ko, K.-K. Cheung, *Inorg. Chem.* **2001**, *40*, 571.

[10] Y. Hasegawa, T. Nakagawa, T. Kawai, *Coord. Chem. Rev.* **2010**, *254*, 2643.

[11] J. Fan, M. Hu, P. Zhan, X. Peng, *Chem. Soc. Rev.* **2013**, *42*, 29.
